# A scanning flow cell approach for generation of electrocatalyst material libraries *via* electrodeposition

**DOI:** 10.1039/d6ta05051d

**Published:** 2026-09-14

**Authors:** Jiahua Zhou, Michael Buchhorn, Ahmad Tirmidzi, Andreas Hutzler, Mingjian Wu, Erdmann Spiecker, Karl J. J. Mayrhofer, Matthias Arenz, Dominik Dworschak

**Affiliations:** a Forschungszentrum Jülich GmbH, Helmholtz Institute for Renewable Energy Erlangen-Nürnberg Cauerstr. 1 91058 Erlangen Germany j.zhou@fz-juelich.de matthias.arenz@unibe.ch d.dworschak@fz-juelich.de; b Department Chemical and Biological Engineering, Friedrich-Alexander-Universität Erlangen-Nürnberg Immerwahrstr. 2a 91058 Erlangen Germany; c Department of Chemistry, Biochemistry and Pharmaceutical Sciences, University of Bern Freiestrasse 3 Bern 3012 Switzerland; d Institute of Micro- and Nanostructure Research (IMN) and Center for Nanoanalysis and Electron Microscopy (CENEM), Department of Materials Science and Engineering, Friedrich-Alexander-Universität Erlangen-Nürnberg Cauerstr. 3 91058 Erlangen Germany

## Abstract

Multi-element material systems offer great property-tailoring possibilities for electrocatalysis. Experimental exploration of multi-dimensional composition spaces requires high-throughput methods for targeted synthesis and testing. We present a scanning flow cell setup that can synthesize material libraries of multi-component alloys *via* electrodeposition, test the electrocatalytic activity of the samples immediately after synthesis, and measure the stability under electrocatalytic conditions with online dissolution monitoring. Using this setup, we explored the Au–Pd–Pt ternary space with 45 samples and demonstrated autonomous exploration guided by Bayesian active learning. The oxygen reduction activity of Au–Pd–Pt alloys exhibits a smooth relationship with composition, decreasing with increasing gold fraction. The stability shows an opposite trend, leading to an inverse relationship between stability and activity.

## Abstract 2

Automated electrodeposition and activity–stability screening of Au–Pd–Pt nanoparticle libraries for ORR catalysis.

## Graphical abstract

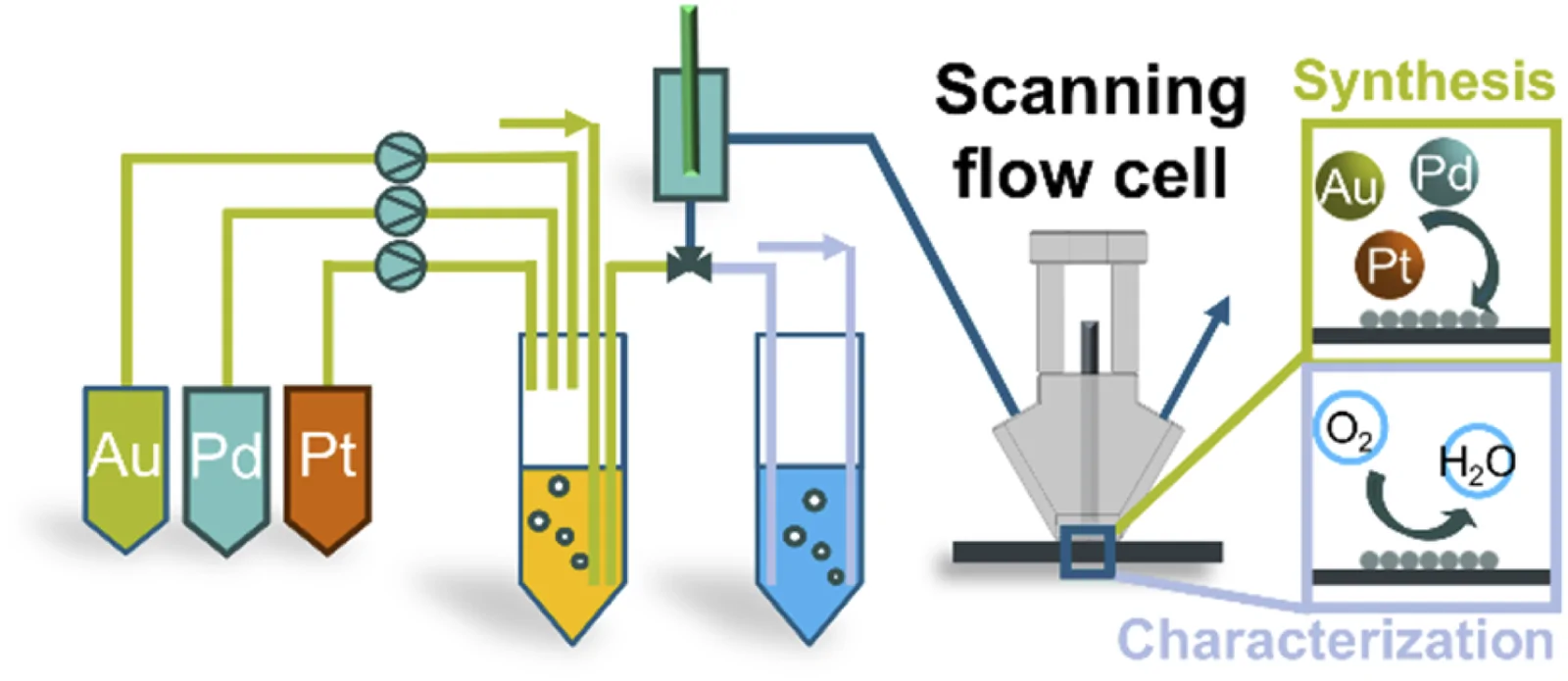

## 1 Introduction

Multinary materials such as high-entropy alloys (HEAs) have potential as catalysts in various electrochemical reactions, such as water splitting reactions, fuel cell reactions, and CO_2_ reduction.^1–4^ Early research on HEA emphasized alloys with (near-) equiatomic composition;^5–7^ however, the properties of a multi-component alloy are not only dictated by the kind of element that constitutes it, but also by the ratios of each component.^8,9^ Both the fundamental understanding of the material system and the search for new materials require investigation of compositions beyond the near-equiatomic region.^10–12^ As the number of constituents increases, the composition space grows combinatorially with a vast amount of possible compositions (Table S1). Therefore, a high-throughput experimental procedure is desired for the analysis of composition–property relationships within a multinary material system.^13^

State-of-the-art high-throughput fabrication methods of material libraries include inkjet printing and co-sputtering.^8^ Inkjet printing produces an array of precursor solution mixtures that usually require calcination to form solid materials. This requirement hampers its throughput and restricts its application to metal oxides.^14,15^ Co-sputtering produces a thin film library with continuously varying compositions but relies on physical vapor deposition conditions that may produce metastable materials under ambient conditions.^12^ Furthermore, targeted synthesis of specific compositions is challenging for co-sputtering, especially toward the faces of the composition space.^9,16,17^ As those compositions often still contribute to the Pareto hypervolume in an HEA system,^18–20^ it is important to also reach the binary to quaternary subsystems of an HEA composition space, *i.e.*, multiple-principal element alloy (MPEA) systems.

Electrodeposition is a versatile method to synthesize alloys from a wide range of available elements including most of the metals from groups 6 to 15.^21^ Alloys from electrodeposition can be either iron-group-based^22,23^ or noble-metal-based,^24,25^ where multiple elements are deposited simultaneously under suitable electrochemical conditions. The morphology of the deposited material can be controlled by the reaction conditions, from homogeneous coatings to various nanostructures as particles, wires or sheets.^24,26–30^ While the relative fractions of elements in the alloys can be altered using deposition parameters,^24,31,32^ the precursor mixture offers the most direct control over the alloy composition.^25,33,34^ This makes electrodeposition ideal for the synthesis of multi-component alloys with targeted compositions.

Besides static or stirred cells, electrodeposition has also been performed in flow cells, where the flow of electrolyte helps sustain mass transport and improve uniformity of the deposit.^33,35–37^ The introduction of different precursor solutions can be computer-controlled, showing the potential to automate the deposition process.^38^

A scanning flow cell (SFC) provides a convenient platform for high-throughput electrochemical characterization.^39,40^ It can scan over many samples in a material library, testing their electrochemical activity. At the same time, the constantly flowing electrolyte in the cell can be analyzed with online analysis, such as electrochemical real-time mass spectrometry (EC-RTMS) and inductively coupled plasma mass spectrometry (ICP-MS), achieving high-throughput activity and stability testing of material libraries.^41–43^

Bayesian optimization can accelerate the discovery of materials by iteratively suggesting the next investigation target based on existing information on the composition–property relationship. Multiple objectives, such as activity, stability, entropy and cost, can be incorporated into the Bayesian framework to search for Pareto-optimal compositions.^44–47^ Using multi-objective Bayesian optimization, Au, Pd, and Pt were found by calculation to be critical contributors to the Pareto front of oxygen reduction reaction (ORR) catalysts in HEA systems.^20^

In this work, we extended the functionality of the SFC from characterization of existing samples to synthesis of new material libraries. We developed an SFC setup that is capable of electrodepositing alloys with targeted compositions and automated it using Bayesian optimization. With this setup, we synthesized material libraries covering the complete composition space of Au–Pd–Pt alloys, tested their catalytic activity for the ORR, and established the stability–activity relationship within the ternary composition space. To our knowledge, no platform has previously combined targeted alloy electrodeposition, immediate electrocatalytic characterization, and Bayesian-optimization-guided exploration within a single scanning flow cell setup.

## 2 Experimental

### 2.1 Electrochemistry

Electrochemical experiments are conducted with a Gamry 620 potentiostat. The electrochemical cell is 3D-printed from stereolithography resin (Formlabs, Clear V4) (Fig. S1). The reference electrode (RE) is a leakless miniature Ag/AgCl electrode (eDAQ). The counter electrode (CE) is a glassy-carbon rod (*ø* 3 mm, SIGRADUR, HTW). The working electrode (WE) is a glassy-carbon plate (50 mm × 50 mm, SIGRADUR, HTW), which is renewed by polishing and then rinsed with isopropanol and deionized water before electrodeposition.

#### 2.1.1 Electrodeposition

The precursor solutions contain 2.5 mM of HAuCl_4_, (NH_4_)_2_PdCl_4_, or (NH_4_)_2_PtCl_6_, and 0.1 M of NaClO_4_. The precursor solutions are mixed in varying ratios across different individual experiments and bubbled with Ar. The flow cell is flushed with the precursor mixture for 120 s at open circuit potential (OCP). Electrodeposition is carried out under a constant flow of 0.4 mL min^−1^. The electrochemical protocol is adapted from Gautam *et al.*^25^ Potential is pulsed between 0.75 and −0.45 V *vs.* RE, each half-cycle lasting 0.015 s. The electrodeposition stops after 120 s, and the remaining solution in the cell is pumped out. The sample is rinsed with DI-water after electrodeposition.

#### 2.1.2 Electrochemical characterization

All electrochemical measurements are carried out in 0.1 M HClO_4_ saturated with O_2_, under a constant flow of 0.4 mL min^−1^. The potential of the reference electrode is calibrated *versus* a reversible hydrogen electrode (RHE), H^+^ (0.1 M)|H_2_ (1 atm)|Pt before activity measurements.

##### 2.1.2.1 Conditioning

The flow cell is filled with electrolyte at OCP. After four cycles of cyclic voltammetry (CV) between 0.05 V and 1.3 V *vs.* RHE with a scan rate of 100 mV s^−1^, OCP is measured again.

##### 2.1.2.2 Double-layer capacitance

The procedure to determine double-layer capacitance is adapted from Morales *et al.*^48^ (Fig. S11). Five cycles of CV are scanned in the window of ±0.1 V at around OCP, with scan rates of 500, 200, 100, 50, and 20 mV s^−1^. We take the average of the absolute values of anodic and cathodic current densities at OCP:
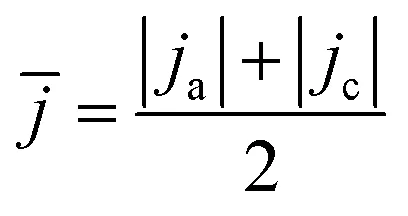
where *j*_a_ and *j*_c_ are anodic and cathodic current densities. The dependence of current density on the scan rate is modeled with the following formula:^49–51^*j* = *k*_1_*ν* + *k*_2_*ν*^0.5^where *j* is current density, *ν* is the scan rate, and *k*_1_ and *k*_2_ represent contributions of capacitive and diffusion current, respectively. The formula is rearranged into a linear form:
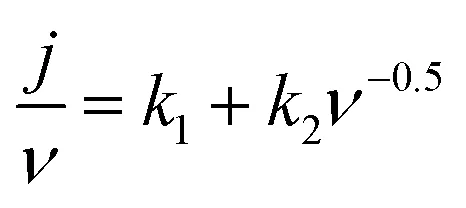
We fit *j̄*/*ν versusν*^−^^0.5^ with linear regression, and the intercept *k*_1_ with a unit of mF cm^−2^ is taken as the specific double-layer capacitance. The mean absolute difference of double-layer capacitance measurements was 0.10 mF cm^−2^ (Fig. S12).

##### 2.1.2.3 ORR activity

Linear sweep voltammetry (LSV) is performed from 1.1 to 0.3 V *vs.* RHE with a scan rate of 10 mV s^−1^. The obtained polarization curves are normalized by division by the double-layer capacitance of each sample. ORR activity is defined as the overpotential (relative to 1.229 V *vs.* RHE^52^) where the specific cathodic current reaches −0.50 mA mF^−1^ or the current density reaches −0.10 mA cm^−2^ (Fig. S9). Measurements where a bubble partially (2 samples) or completely (5 samples) obstructed the flow channel were excluded (Fig. S10). Each composition is measured once. The standard deviation of the ORR activity measurement was found by repeated experiments to be 36 mV (Fig. S9f).

### 2.2 Stability study

The dissolution of the elements is recorded using ICP-MS (Agilent 7900). Ion counts of ^105^Pd, ^195^Pt, and ^197^Au are converted to mass fluxes using four-point calibration curves. ^103^Rh (for ^105^Pd) and ^185^Re (for ^195^Pt and ^197^Au) are used as internal standards. The electrolyte is O_2_-saturated HClO_4_ (0.1 M) with a flow rate of 0.3 mL min^−1^.

The electrochemical protocol, during which dissolution signals are recorded, is listed in Table S4. The core of the stability study is an accelerated stress test (AST), *i*.*e.*, potential pulsing between 0.6 and 1.4 V *vs.* RHE, with 3 s on each step, for a total time of 20 min.^53–55^ The potential range was chosen to maximize the change of activity through the AST. The total dissolution amount during the AST was recorded and normalized by the total mole fraction of the three metal elements determined by SEM-EDX to represent stability. This AST protocol is flanked by two identical test procedures for the ORR activity (as in Section 2.1.2) to test the change in activity. Immediately after the second activity test, CV is performed between 0.3 and 1.4 V at 10 mV s^−1^, in order to acquire the potential-dependent dissolution behavior of the elements (Table S5).^56^ Between each section, at the beginning, and at the end of the protocol, OCP is held to allow the ICP-MS signal to settle to a steady state.

### 2.3 SEM-EDX analysis

The scanning electron microscopy (SEM) images are recorded on a Tescan Vega 3 with an acceleration voltage of 10 kV and a working distance of 15 mm, using a secondary electron (SE) detector. The energy dispersive X-ray (EDX) signals are recorded with an EDAX AMETEK ELEMENT detector. The signals are averaged from a grid scan inside a 3 mm × 3 mm square. The signals are deconvolved with the eZAF method using the EDAX TEAM software. On each spot, the measurement is repeated six times (30 s each). A confidence region is estimated from the repeated measurements, and the geometric average of the results is taken as the mean composition (Fig. S7b).

The quantification results from EDX were cross-validated with those of destructive elemental analysis by ICP-MS. The two methods show general agreement over the fractions of Au, Pd and Pt, but EDX has a higher sensitivity for Pd/Pt and a lower sensitivity for Au than ICP-MS (Fig. S8a).

We apply consistent analysis settings on every sample and report the compositions of all samples as the EDX quantification suggests. However, due to quantification uncertainty and, in particular, similar emission wavelengths of Pt and Au (Pt Mα_1_ of 2.050 keV and Au Mα_1_ of 2.123 keV),^57^ a small amount (up to 7%) of every element is always detected, even in samples from only one or two precursor metals. For clarity, we refer to the samples derived from only two precursors by the relative compositions between their precursor elements (*e.g.* Pd_43_Pt_57_). We refer to the samples derived from a single precursor not as ‘pure’ gold, *etc.*, but as single-metal samples.

### 2.4 Bayesian active learning

We use the Gaussian Process (GP) to model the precursor-nanoparticle composition relationship (composition model) and the nanoparticle composition–activity relationship (activity model). The GP model defines a distribution over functions, based on a prior and observed data.^58^ The covariance of random variables in the GP is defined using a covariance function (kernel). The kernel used in both models is a Matérn kernel with ν = 1.5.
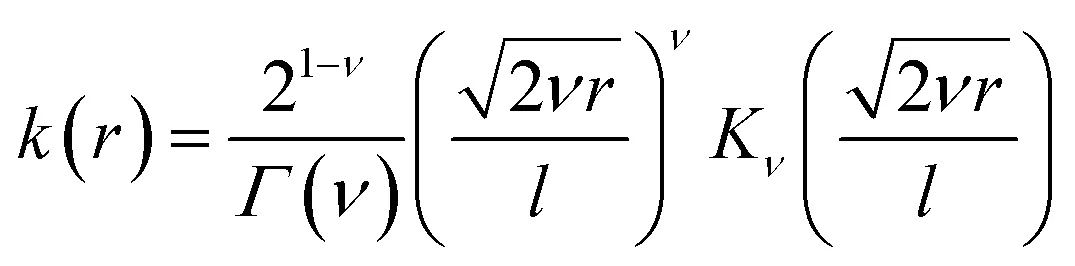


The Matérn kernel represents a generalization of the radial basis function (RBF) with higher roughness, where *ν* and *l* are positive parameters and *K*_*ν*_ is a modified Bessel function.^58^

For the composition model, the compositions are transformed by a centered log-ratio transformation.^59^ For the activity model, the data are not transformed, and thus the Matérn kernel is multiplied by a constant kernel. The constant and the characteristic length scale *l* are hyperparameters fitted during model training.

Because of limitations of the EDX-characterization, all possible EDX-compositions form a subset of the ternary simplex (Section 2.3). The input of the Bayesian procedure is restrained inside this EDX-subset (Fig. S16a) to avoid unrealistic suggestions (like 100% Au as EDX-composition).

## 3 Results and discussion

### 3.1 Scanning flow cell setup

The setup features a liquid selection unit at the inlet of a SFC that switches the fluid depending on the operation mode of the setup (Fig. 1a). In the synthesis mode, three independent channels of a peristaltic pump push three different precursor solutions into a mixing vial. The running time of each channel is individually set in order to mix the precursor solutions in a specific ratio. Argon bubbles from a gas line facilitate mixing of solutions and remove oxygen from the resulting solution. The precursor mixture is pumped through the flow cell while applying potential pulses, whereby nanoparticles are deposited.

**Fig. 1:**
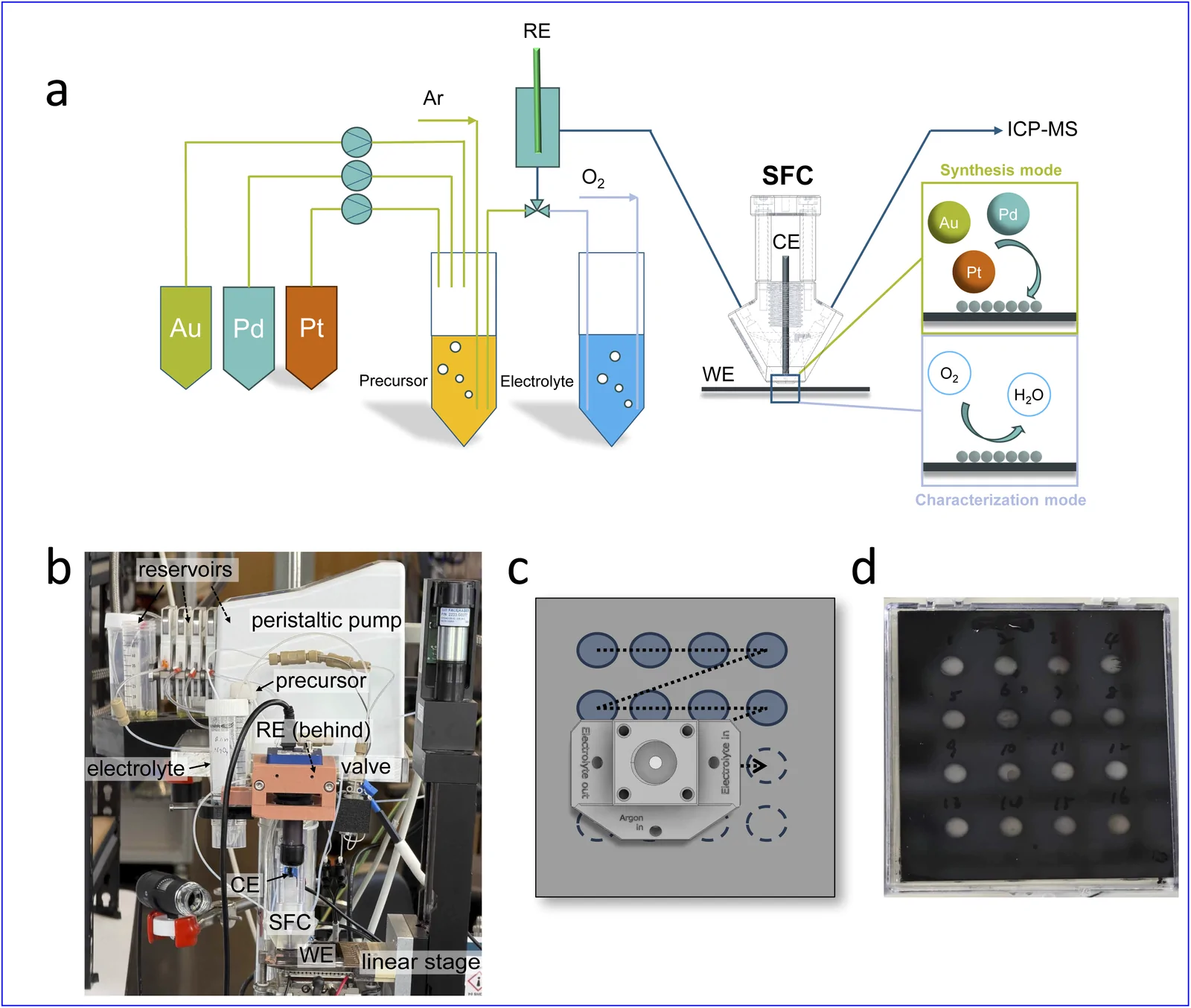
(a) The experimental setup. Green lines are active in the synthesis mode, light blue lines are active in the characterization mode, and dark blue lines are shared by both modes. Au, Pd and Pt represent the corresponding precursors. (b) Photo of the setup. (c) Schematic showing the scanning movement of the cell over the substrate. (d) Photo of a material library with 16 samples. WE: working electrode, CE: counter electrode, RE: reference electrode, SFC: scanning flow cell, and ICP-MS: inductively coupled plasma mass spectrometry.

In the characterization mode, perchloric acid as electrolyte is pumped through the flow cell, in order to test the electrocatalytic activity of the samples in the ORR. Furthermore, the outlet of the flow cell can be coupled to an ICP-MS for real-time analysis of the dissolution of the samples under electrochemical conditions.

Inside the SFC, the WE comes into contact with the electrolyte within an elliptical contact area (15 mm^2^). An O-ring seals around the contact area, preventing leakage of liquid out of the cell. The flow channels in the SFC form a V-shape (Fig. S1), similar to the geometries used in other studies.^33,39,41,42^ In this geometry, the current flux is not uniformly distributed on the contact area of the WE due to diffusion limitations.^60^ Usually the CE is placed on one side of the channels, introducing an asymmetry that would also cause uneven coverage of the substrate by deposited particles. In our setup, the CE is placed perpendicularly above the opening of the SFC instead of outside the cell or in one of the channels, and the lower surface of the CE projects symmetrically onto the contact area. This arrangement improved homogeneity of the deposited sample by providing a symmetric electric field between the CE and WE (Fig. S2). The RE is placed outside the SFC, in the flow path of the electrolyte.

The WE is mounted on a three-axis stage (Fig. 1b), so that the cell can scan over the substrate. As the testing on one sample is finished, the cell detaches itself from the WE and moves over to the next designated position for the synthesis and testing of a new sample, producing an array of samples (Fig. 1c). Up to 16 samples can be deposited onto a substrate during an unattended session, forming a material library (Fig. 1d).

### 3.2 AuPdPt material libraries

We synthesized 45 compositions of Au–Pd–Pt alloys from precursor mixtures at every grid point spaced 12.5% apart, inside the Au–Pd–Pt ternary composition space (Fig. 2a). The composition of the nanoparticles was determined by SEM-EDX analysis. A systematic shift was observed between precursor compositions and nanoparticle compositions, in the direction towards Au (Fig. 2b). A GP model trained to predict EDX-composition and precursor-composition correspondences also reflects this tendency (Section 3.3, Fig. 4a). This shift is expected, due to the reduction potential of AuCl_4_^−^ being the highest among the precursor ions, in the range of Cl^−^ concentration during electrodeposition (Table 1). Thus, at the same deposition potential, the overpotential of the reduction of AuCl_4_^−^ to Au would be the most negative, which leads to a higher reaction rate according to the Tafel equation. The higher deposition rate of gold is also reflected by the higher loading at compositions containing more gold (Fig. 2c). On the other hand, if the reduction is controlled by mass-transfer, the diffusion-limited deposition current would be determined by the diffusion coefficient according to the Levich equation, where we would expect similar rates between Au^3+^ and Pt^4+^.^25^ This suggests that a complete mass-transfer limitation is unlikely under our experimental conditions.

**Fig. 2:**
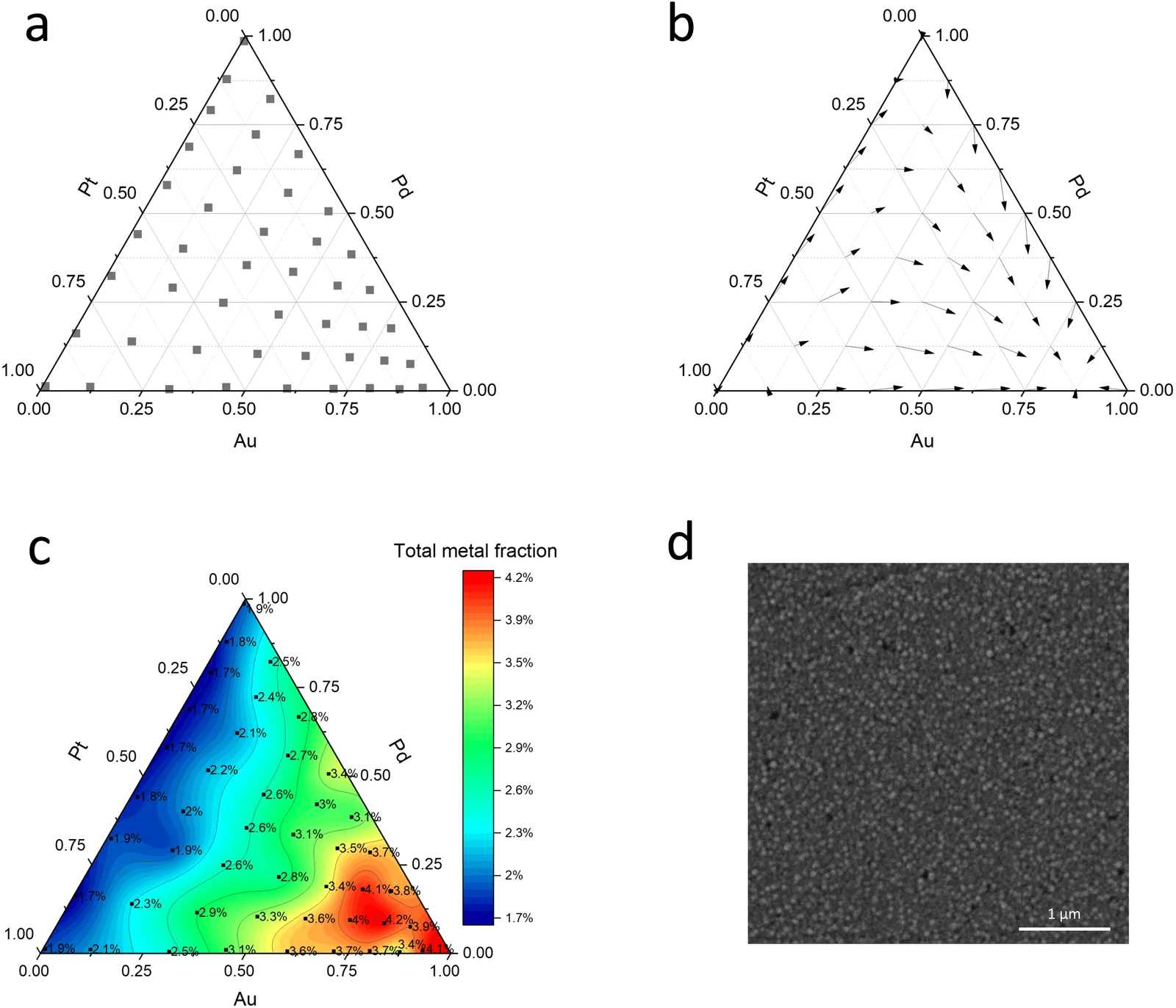
(a) Composition of nanoparticles determined by SEM-EDX. (b) Comparison between precursor and EDX compositions. Each vector points from the precursor composition to the EDX composition of a sample. (c) Percentage of the total EDX-fraction of Au, Pd and Pt, relative to the sum of C, O, Au, Pd and Pt. (d) Exemplary SEM image of Au_40_Pd_36_Pt_24_.

**Table 1:** Standard reduction potentials of the precursor ions^61^ and the range of reduction potentials under real deposition conditions (Table S2)

Reaction	*E*°/V	*E*_real_/V_RHE_
[AuCl_4_]^−^ + 3e ⇌ Au + 4Cl^−^	1.002	1.193–1.304
[PdCl_4_]^2−^ + 2e ⇌ Pd + 4Cl^−^	0.591	0.878–1.044
[PtCl_6_]^2−^ + 4e ⇌ Pt + 6Cl^−^	0.718	0.933–1.058

The deposited alloys formed nanoparticles of a 30–110 nm mean diameter (Fig. 2d and S4). The particle size and morphology can be altered using deposition parameters, such as seed concentration, synthesis time and duty cycle during pulsing.^25,62,63^ For the purpose of high-throughput screening, we did not tailor individual protocols for different compositions but adjusted the protocol to achieve a uniform coverage of the substrate (Fig. S3) and applied this condition to all samples to produce nanoparticles within the above-mentioned size range. This range exceeds the typical size (up to 10 nm) where variation in particle size would significantly impact their electrochemical active surface area (ECSA) and specific ORR activity.^34,64,65^ Transmission electron microscopy (TEM) and X-ray diffraction (XRD) confirmed a ternary alloy with uniform distribution of the elements and an fcc structure from an equimolar mixture of the precursors (Fig. S5 and S6).

We measured the ORR activity of the material libraries with LSV scans in O_2_-saturated electrolyte (Fig. 3a and b). Since the mass loading differs between compositions (Fig. 2c) and there are uncertainties regarding the mass loading determination by either EDX or ICP-MS (Fig. S8b and c), normalization of the activity by the ECSA is necessary. However, a universal method for the measurement of the ECSA across the Au–Pd–Pt composition space poses a challenge. The ECSA of platinum can be measured with hydrogen underpotential deposition (*H*_UPD_),^66,67^ but on palladium, the adsorbed hydrogen diffuses into the bulk^68^ and the adsorption on gold is negligible,^69^ excluding these metals from the *H*_UPD_ method. The CO-stripping method is also well established for Pt, but the saturated coverage on Pd is uncertain,^70^ while no usage of this method on gold is reported. The surface oxide reduction method has been applied to all three elements,^69–73^ but in our acidic electrolyte, we did not observe the oxide reduction peak of gold or neighboring compositions (Fig. S9a). To accommodate all compositions, we used the double-layer capacitance (*C*_dl_) method^48^ to measure the ECSA. The measured double-layer capacitance could be converted to surface area using the specific capacitance (*C*_s_) of the material,^48,74^ but the precise value of *C*_s_ of a material depends on its morphology.^75,76^ Because the deposited particles of binary or ternary compositions do not have a uniform morphology, their *C*_s_ remains uncertain, and we report the *C*_dl_ itself to represent the ECSA (Fig. 3c).

**Fig. 3:**
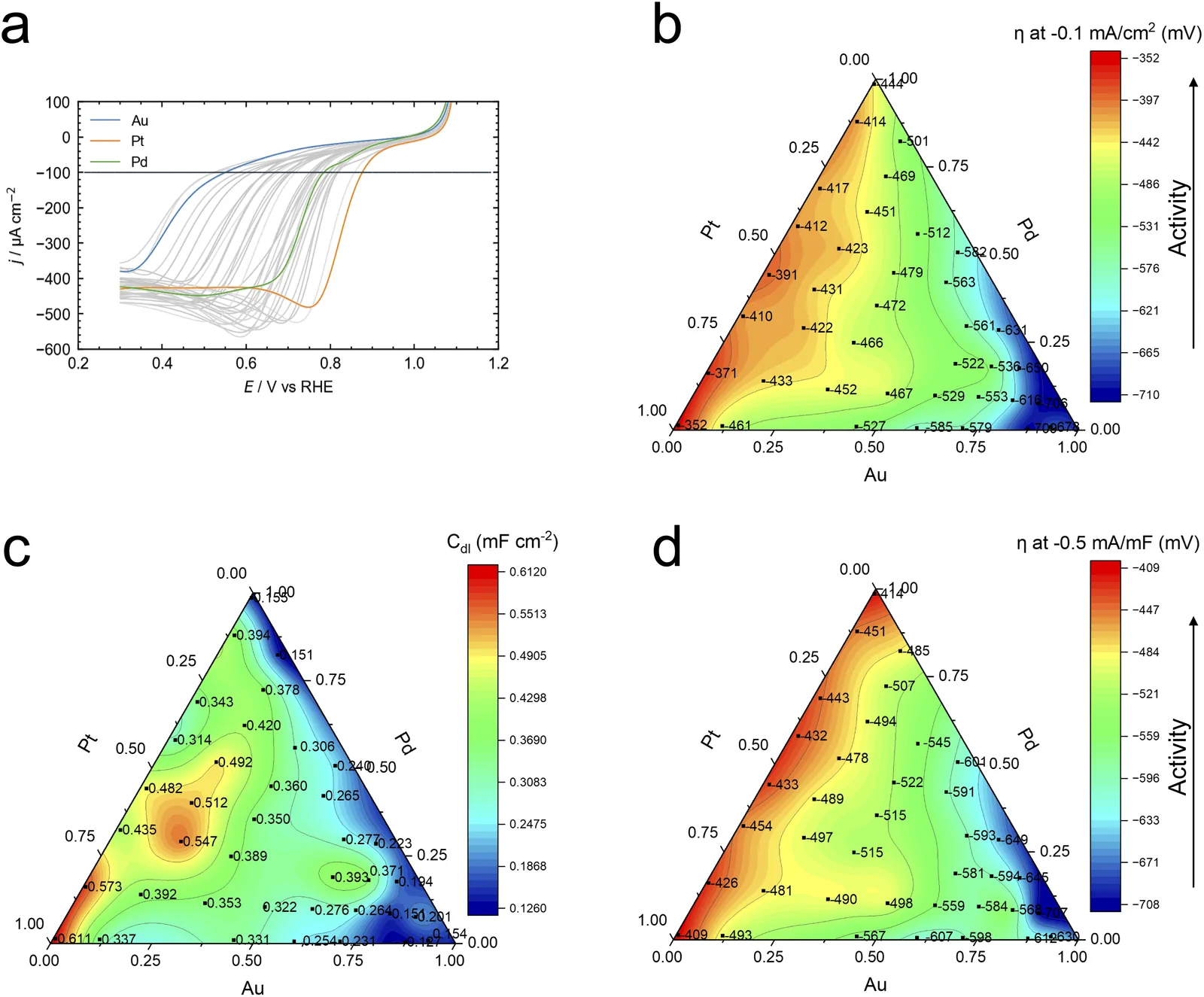
(a) LSV scans of the material libraries. The curves of the single-metal samples are highlighted. Dashed lines indicate the current density at which the potential is taken as ORR activity. (b) ORR activity as the overpotential at −0.1 mA cm^−2^. (c) Double-layer capacitance in mF cm^−2^. (d) *C*_dl_-normalized ORR activity as the overpotential at −0.5 mA mF^−1^.

The *C*_dl_-normalized ORR activities of the alloys are plotted as a function of their composition in a ternary graph (Fig. 3d), which share a similar overall trend with the geometric ORR current (Fig. 3b). Compositions with the highest activities are found from the series of Pd–Pt binary alloys. As the fraction of gold increases, the ORR-activity of the alloy decreases almost monotonically, showing that the activity of AuPdPt ternary alloys can be smoothly tuned by their composition, just as in the Au–Pt and Au–Pd binary subspaces.^77–79^ The trend of ORR activity agrees with the prediction from the volcano curve, where the oxygen binding energies of platinum and palladium sit near the top of the volcano, while the reduction of O_2_ on gold is limited by its weak oxygen-binding ability.^80,81^

### 3.3 Bayesian active learning

With the ability to target a specific composition and get feedback on its activity, our setup provides a proper experimental platform for autonomous exploration on multiple-component alloys. We used Bayesian approaches to synthesize a material library with minimal human intervention that aims to reflect the trend of activity in the Au–Pd–Pt composition space.

In a Bayesian approach, the position of the next sample is directed by the maximum value of the acquisition function.^82^ We chose the uncertainty of the model prediction as the acquisition function and performed Bayesian active learning, as we aimed fully for exploration of the composition space. Since the distribution of activity in the Au–Pd–Pt ternary space is relatively smooth, the overall trend can be constructed from a practical number of samples, from which the optimum of the activity can be readily inferred. For alloy systems with higher dimensionality and more complexity, an acquisition function for a balance between exploration and exploitation, *e.g.* Probability of Improvement (PI) or Expected Improvement (EI), can be selected for the Bayesian optimization of activity, to search for optimal catalysts more efficiently.^44,83,84^

We implemented a GP model as the main model for the relationship between EDX-composition and activity (the activity model). We also used correspondences between precursor- and EDX-compositions obtained from previous experiments (Section 3.2) to train a GP model (the composition model) that predicts the EDX-composition for every possible precursor-composition and *vice versa* (Fig. 4a and S16), as a surrogate model for the SEM-EDX analysis that was not available immediately.

**Fig. 4:**
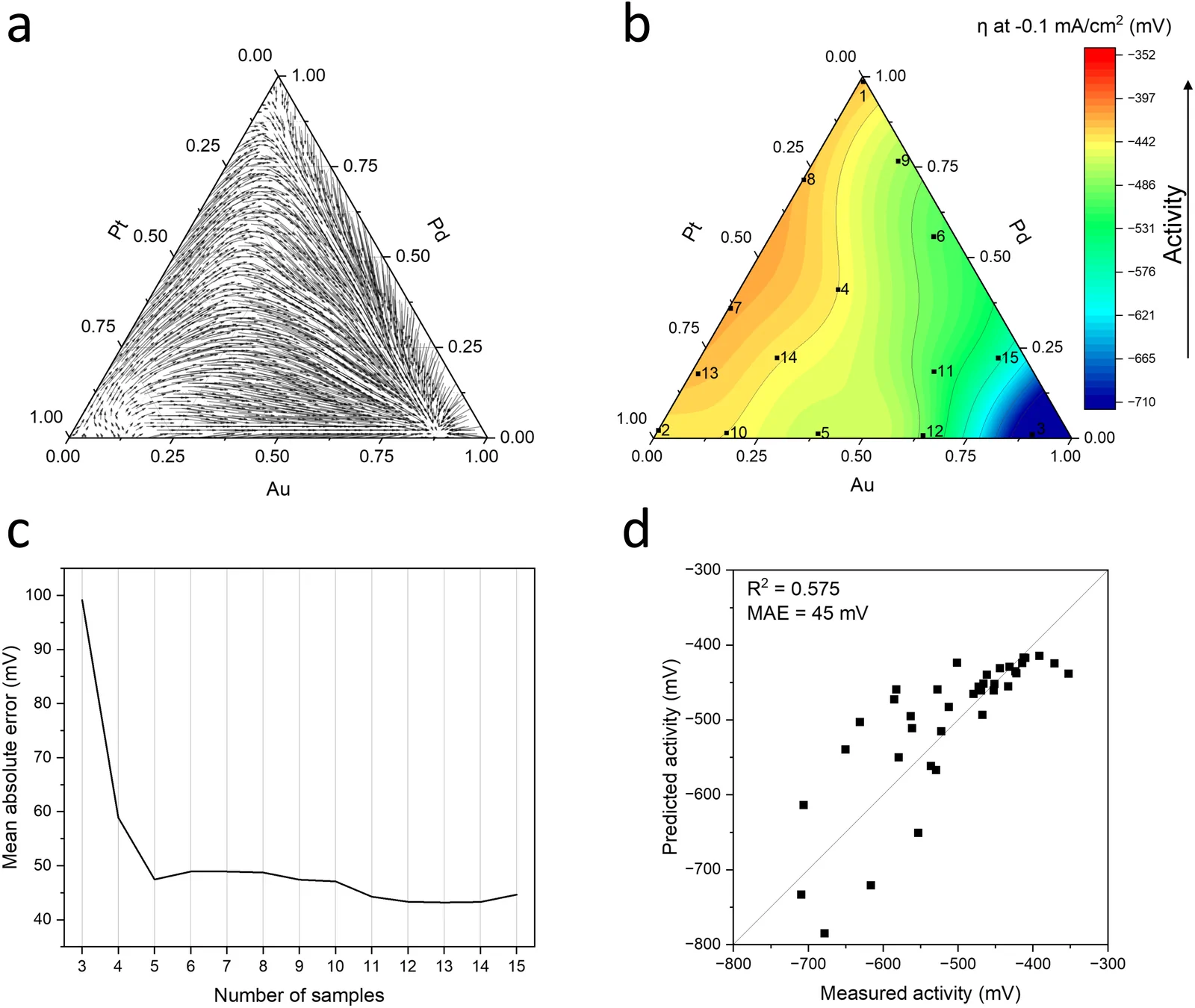
(a) Prediction of the composition model. Each vector points from a precursor-composition to the corresponding EDX-composition. (b) Prediction of the activity model, plotted in the EDX-composition simplex. The number on each composition is the order of suggestion by the model. The color bar conforms with Fig. 3b. (c) Prediction error of the activity model on the previous dataset (Section 3.2) *versus* the number of samples. (d) Performance of the activity model with all 15 samples, tested against the previous experimental dataset. The solid line represents perfect prediction. *R*^2^: coefficient of determination. MAE: mean absolute error.

The workflow of the experiment is shown in Scheme 1. The three single-metal compositions at the vertices of the composition space are first synthesized, and their ORR-activities serve as the input for the process. We fit the activity model and use it to evaluate the acquisition function at every point inside the EDX-composition grid. The composition with the highest acquisition value (in this case, the uncertainty of prediction) is chosen as the target for the next synthesis. The precursor-composition needed to get this EDX-composition is predicted by the composition model, and the protocol for the next synthesis is generated. The setup proceeds to synthesize the next sample and then its activity is analyzed and fed back into the activity model to improve its prediction. The cycle repeats until we have obtained 15 samples.

**Scheme 1:**
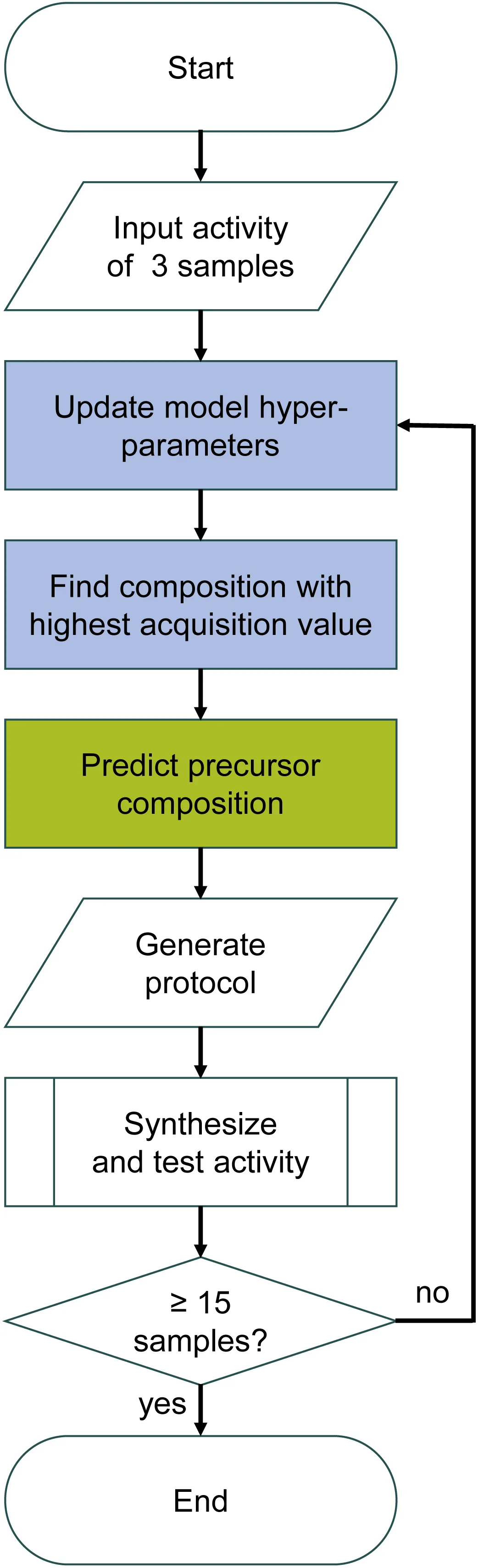
Workflow of the Bayesian-guided experiment. Implementation of GP models is indicated by background color, blue: activity model and green: composition model.

The activity model predicted the previously observed trend of ORR activity that decreases as the fraction of gold increases (Fig. 4b and compare Fig. 3b). We used the dataset from Section 3.2 to evaluate the performance of the activity model as more samples are added to the model. The composition–activity relationship in the AuPdPt ternary space can be decently modelled with a small number of samples selected with Bayesian active learning (Fig. 4c and d). The distribution of activity is relatively smooth and monotonic, while the extreme values lie on the edge and vertex of the simplex. With the information of the three vertex samples and two more samples from the inside, the GP model can already establish the overall trend.

### 3.4 Stability–activity relationship

Because the stability measurement has a longer duration than the activity measurement (∼45 min *vs.* ∼5 min), reducing the number of experiments is therefore particularly important. Following the suggestion from Bayesian optimization, a grid covering the ternary simplex would be efficient for exploration, and we conducted stability measurements on a material library with 15 compositions, similarly spaced as in Section 3.3. We recorded the dissolution of the three elements during an accelerated stress test (AST) and compared the ORR activity before and after the AST to assess the stability and performance change of the samples.

The single-element samples of palladium and gold show the greatest and the least amount of dissolution respectively, among all samples. The total dissolved amount normalized by mass loading generally decreases as the proportion of gold in the alloy increases (Fig. 5a). The presence of gold also inhibits the dissolution of other elements (Fig. S13).

**Fig. 5:**
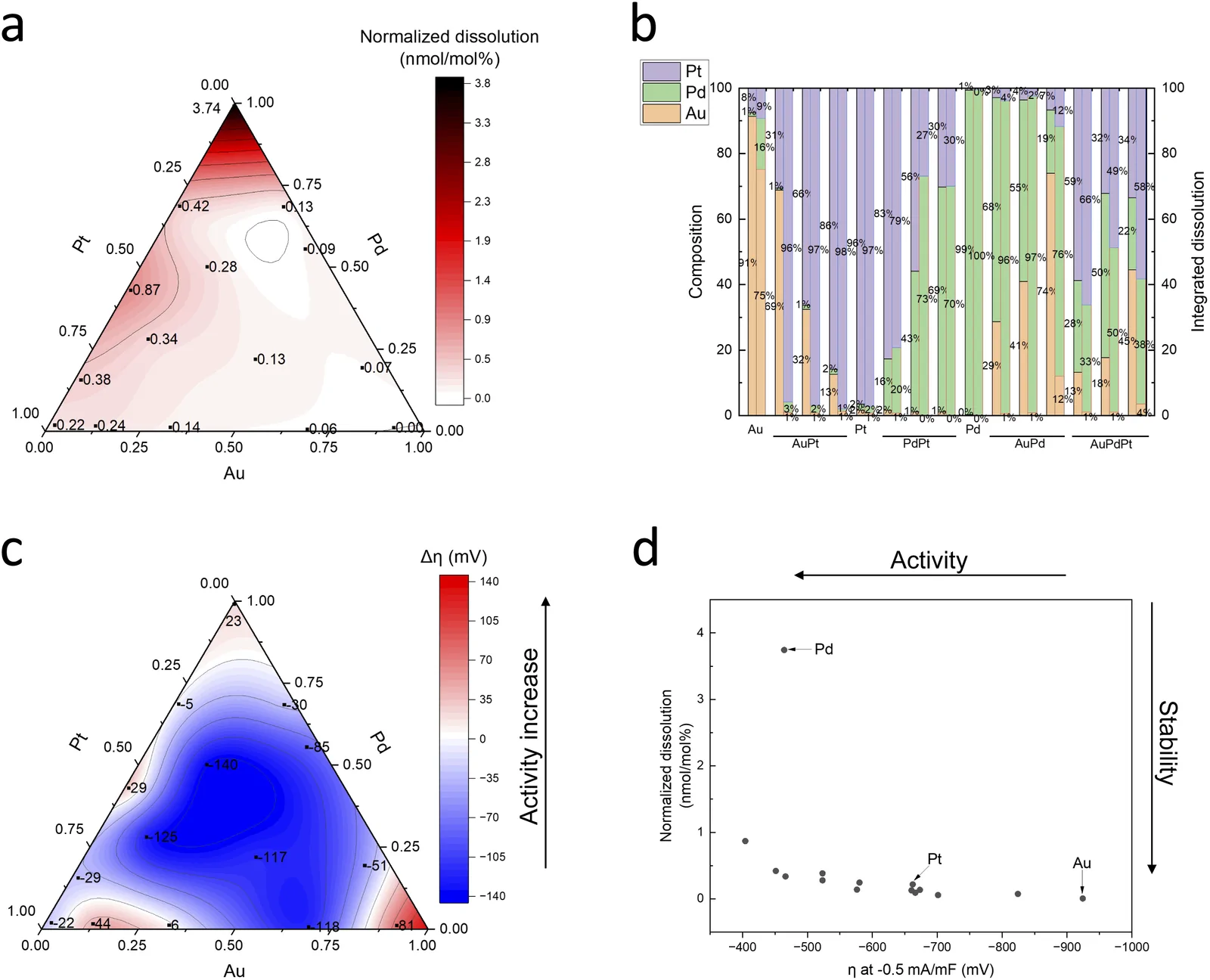
(a) Total dissolution normalized by the EDX-metal-fraction, plotted in the composition space. (b) Bulk and dissolved composition. Within each column pair, the left column is the EDX-composition of a sample and the right column shows the proportion of the elements in the total dissolved amount. Detailed data are in Table S5. (c) Change in overpotential after the AST. (d) Normalized dissolution as a function of ORR activity. The left-bottom corner represents the ideal catalyst.

Further analysis of the share of elements in binary and ternary alloys from the total dissolved amount reveals a selective dissolution process which explains the change in activity after the AST (Fig. 5b and c):

• The portion of gold in dissolution is much less than that of gold in bulk composition.††The single-element gold sample seems to have a considerable share of Pd and Pt in its dissolution, because the dissolution of gold was so low that it had the same magnitude as the background signal of Pd and Pt, up to 10^−2^ nmol (Table S5).

For binary or ternary alloys containing gold, this would cause an enrichment of gold atoms on the surface of the nanoparticles after the AST while the initial surface was rather depleted of gold (Fig. S5h), resulting in a general decrease of activity in those regions.

• The proportion between palladium and platinum in dissolution roughly corresponds to that of their bulk composition.

Together with the similar ORR activities of palladium and platinum, the activity of Pd–Pt binary alloys remained similar before and after the AST.

The single-element gold sample experienced the greatest *increase* of activity after the AST by 81 mV. While gold had very low dissolution compared to the other compositions, its *C*_dl_ increased by 64%, indicating that the AST caused the surface gold atoms to restructure rather than dissolve, which activated the sample.^85,86^ Potential cycling can also form an oxide layer on gold, enhancing its ORR activity.^87,88^ An activation by the AST is also evident on Pd_43_Pt_57_ (Fig. S14).

The stability, represented as the total amount of dissolved atoms, and the activity, represented as the *C*_dl_-normalized ORR-overpotential, show a strong association with each other (Fig. 5d). Except for single-element palladium, all compositions form a curve that dictates an inverse relationship between stability and activity, as reported in many material systems for oxygen evolution or reduction catalysis.^89–93^ While palladium is susceptible to electrochemical dissolution in acidic solution,^94^ simulations have shown a stabilizing effect of a low molar fraction of gold in Au–Pd nanoparticles, and in an experiment, 20 at% Au could reduce Pd dissolution by 67%.^95^ Improved electrochemical stability of Pd–Pt nanoparticles over palladium has also been observed.^94,96^

In terms of multi-objective optimization,^18,97^ almost every composition from the Au–Pd–Pt composition space lies on the Pareto front of activity and stability (Fig. 5d). When the prices of the metals are considered the third objective, the binary alloys Pd_16_Pt_84_, Pd_43_Pt_57_, and Pd_69_Pt_31_ stand out with low costs and high activities; nevertheless, we still encounter a stability–activity trade-off among those compositions (Fig. S15).

## 4 Conclusion

This work presents an SFC-setup for the generation and characterization of multiple-component alloys *via* electrodeposition. The setup can synthesize and characterize the activity of one sample within 15 minutes (Scheme 2), which allowed us to screen the Au–Pd–Pt composition space for ORR activity with 45 samples in two workdays. We found the highest ORR activity among Pd–Pt alloys and the highest stability around the Au-edge, requiring a compromise between activity and stability.

**Scheme 2:**
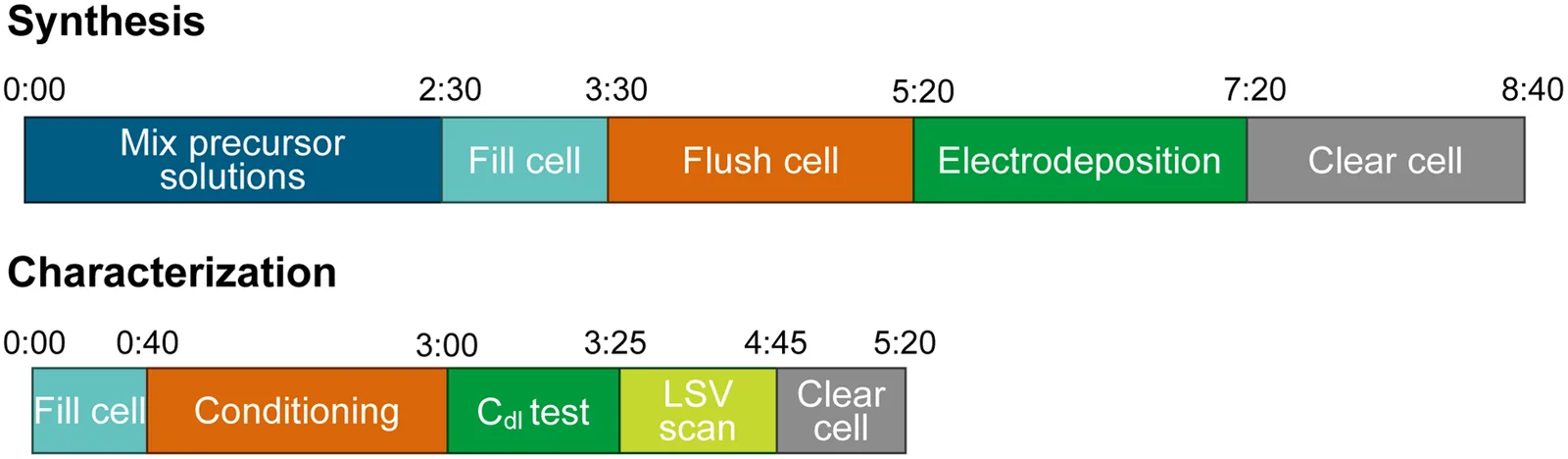
Segmentation of time (min:sec) needed in synthesis and activity characterization of one spot. The preparation of the chemicals, the polishing of the WE and the setup of the SFC require an additional two hours.

The setup is a useful tool for synthesis parameter–performance screening of electrocatalyst, coating or sensor materials with the limitation that the material can be deposited electrochemically from a precursor solution.

The presented workflow demonstrates how autonomous experimentation can be integrated with targeted synthesis and real-time stability characterization to accelerate the exploration of complex electrocatalyst composition spaces. The approach is readily extendable to higher-dimensional alloy systems by adding channels of precursors of compatible elements.^25^ Other objectives, such as stability and cost, can be included in the Bayesian algorithm for multi-objective catalyst optimization.^20^ In conclusion, this work provides an integrated platform for high-throughput exploration of HEA compositional spaces.

## Author contributions

Jiahua Zhou: methodology, formal analysis, investigation, visualization, writing – original draft. Michael Buchhorn: methodology, resources, writing – review & editing. Ahmad Tirmidzi: methodology, software, investigation, writing – review & editing. Andreas Hutzler: investigation, visualization, writing – review & editing. Mingjian Wu: investigation, writing – review & editing. Erdmann Spiecker: resources, writing – review & editing. Karl J. J. Mayrhofer: funding acquisition, writing – review & editing. Matthias Arenz: conceptualization, supervision, writing – review & editing. Dominik Dworschak: conceptualization, supervision, project administration, writing – review & editing.

## Conflicts of interest

There are no conflicts to declare.

## Supplementary Material

TA-014-D6TA05051D-s001

## Data Availability

Additional data related to this work, including the SEM-EDX, electrochemistry and dissolution data, the scripts for data analysis and for Bayesian active learning are available at https://doi.org/10.5281/zenodo.21917949. Technical drawing of the cell, SEM & TEM images, detailed information for the activity and stability measurements, validation of experimental results, discussions on the GP models, and various further data supporting this article have been included in the supplementary information (SI). Supplementary information is available. See DOI: https://doi.org/10.1039/d6ta05051d.
