# Supplementary material for: A scanning flow cell approach for generation of electrocatalyst material libraries via electrodeposition
Source: J Mater Chem A Mater. 2026 Sep 14;14(63):43862–72. doi: 10.1039/d6ta05051d (PMC13632669; doi:10.1039/d6ta05051d)
Supplement: Supplementary file 1 [file TA-014-D6TA05051D-s001.pdf]

Supplementary Information (SI) for Journal of Materials Chemistry A.
This journal is © The Royal Society of Chemistry

Supplementary Information for:

A scanning flow cell approach for generation of electrocatalyst material libraries via electrodeposition

Jiahua Zhou,*[a,b] Michael Buchhorn,[a,b] Ahmad Tirmidzi,[c] Andreas Hutzler,[a] Mingjian Wu,[d] Erdmann Spiecker,[d] Karl J. J. Mayrhofer,[a,b] Matthias Arenz,*[c] Dominik Dworschak*[a]

Contents

1 Size of composition space **4**

2 Scanning flow cell **5**

3 Electrode positioning **6**

4 Calculation of reduction potentials **7**

5 Nanoparticle morphology **8**

6 Transmission electron microscopy **10**

7 X-ray diffraction **12**

---

[a] Jiahua Zhou, Michael Buchhorn, Andreas Hutzler, Karl J. J. Mayrhofer, Dominik Dworschak
Helmholtz Institute Erlangen-Nürnberg for Renewable Energy (IET-2), Forschungszentrum Jülich, Cauerstr. 1, 91058 Erlangen, Germany.
Homepage: `https://www.hi-ern.de/en`

[b] Jiahua Zhou, Michael Buchhorn, Karl J. J. Mayrhofer
Department of Chemical and Biological Engineering, Friedrich-Alexander-Universität Erlangen-Nürnberg, Cauerstr. 1, 91058 Erlangen, Germany.

[c] Ahmad Tirmidzi, Matthias Arenz
Department of Chemistry, Biochemistry and Pharmaceutical Sciences, University of Bern, Freiestrasse 3, Bern 3012, Switzerland

[d] Mingjian Wu, Erdmann Spiecker
Institute of Micro- and Nanostructure Research (IMN) and Center for Nanoanalysis and Electron Microscopy (CENEM), Department of Materials Science and Engineering, Friedrich-Alexander-Universität Erlangen-Nürnberg, Cauerstr. 3, 91058 Erlangen, Germany

E-mail: j.zhou@fz-juelich.de, matthias.arenz@unibe.ch, d.dworschak@fz-juelich.de

8 Composition confidence **13**

9 Composition cross-validation **14**

10 Electrochemical characterization **15**

11 Double layer capacitance measurement **17**

12 Stability measurement **19**

13 Multiobjective optimization **22**

14 Gaussian process models **23**

List of Figures

S1 CAD drawing of the scanning flow cell. 5
S2 Electrode positioning and homogeneity of spot. 6
S3 SEM images of nanoparticles from different synthesis conditions. . . 8
S4 Nanoparticle size from SEM images. 9
S5 TEM analysis of a three-element sample. 10
S6 XRD patterns of a three-element sample. 12
S7 Confidence regions of EDX composition 13
S8 Composition determination by ICP-MS and SEM-EDX 14
S9 Electrochemical characterization during activity measurement. . . . 15
S10 Effect of bubble on LSV measurement. 16
S11 Example of double layer capacitance measurement. 17
S12 Measurement uncertainty of C_{dl} 18
S13 Distribution of relative dissolution for each element. 20
S14 Before-and-afters of the ORR overpotential, ECSA and ECSA-normalized activity. 21
S15 Pareto fronts of the compositions. 22
S16 Outputs of Gaussian Process models. 24
S17 Comparison of sampling methods for GP. 25

List of Tables

S1 Number of possible compositions in composition spaces. 4
S2 Cumulative stability constants and calculated Cl^- concentrations. . . 7
S3 Linear regression between ICP-MS and EDX compositions. 14
S4 Electrochemical protocol for stability study. 19
S5 Composition of the samples tested for stability and their dissolution. 20
S6 Fitted hyperparameters of GP with and without white kernel to represent noise. MAE is measured against a 37-sample ground truth. . 23

1. Size of composition space

Number of constituents	Composition step size				
	12.5%	10%	5%	2%	1%
2	9	11	21	51	101
3	45	66	231	1326	5151
4	165	286	1771	23426	176851
5	495	1001	10626	316251	4598126

Table S1: Number of possible compositions in composition spaces.

The numbers come from binomial coefficients:

$$\binom{d+n-1}{n} = \frac{(d+n-1)!}{n!(d-1)!} \tag{S1}$$

where d is the number of constituents or the dimension of the composition space, and n is the reciprocal of the composition step size.

2. Scanning flow cell

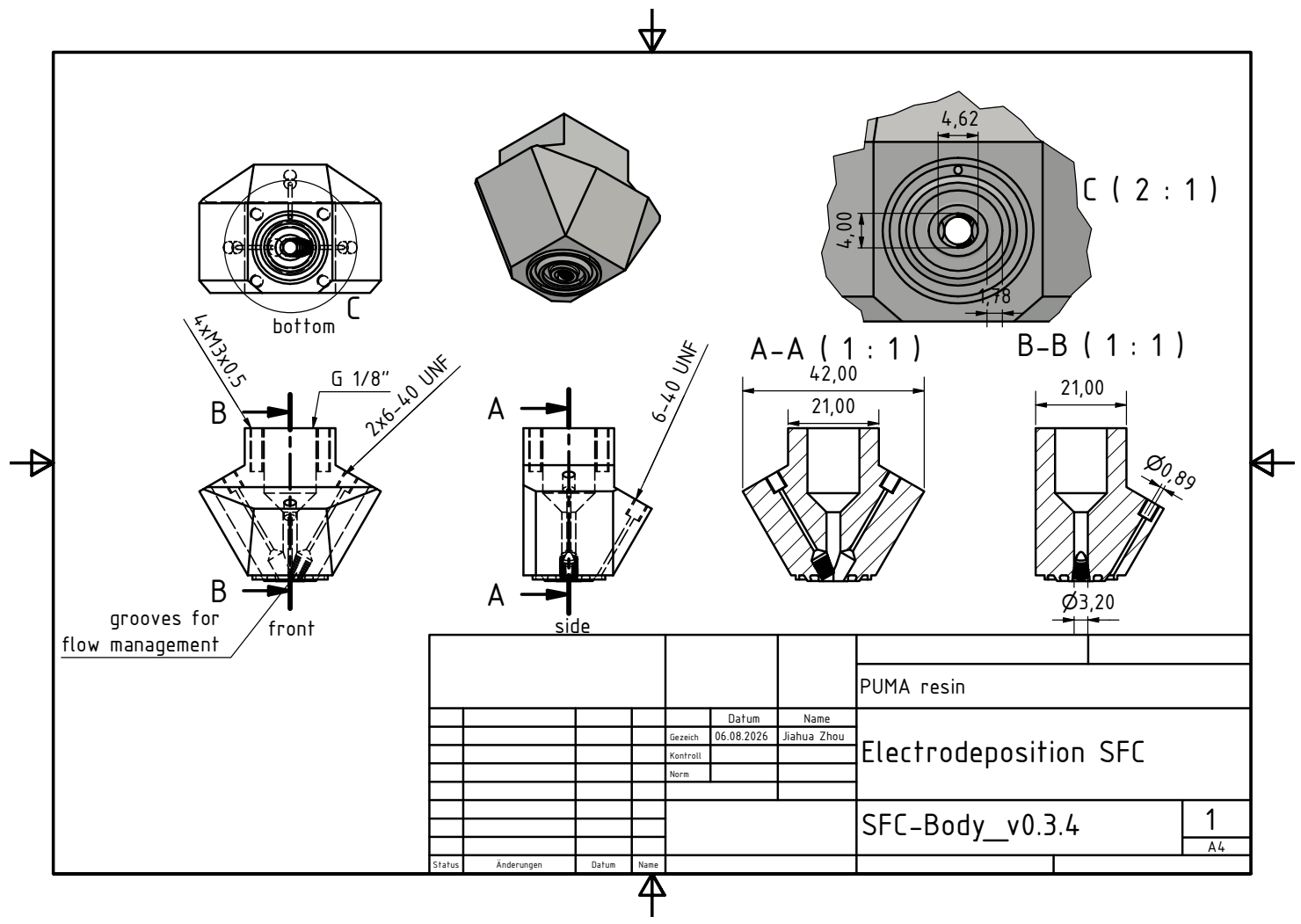

Figure S1: CAD drawing of the scanning flow cell.

3. Electrode positioning

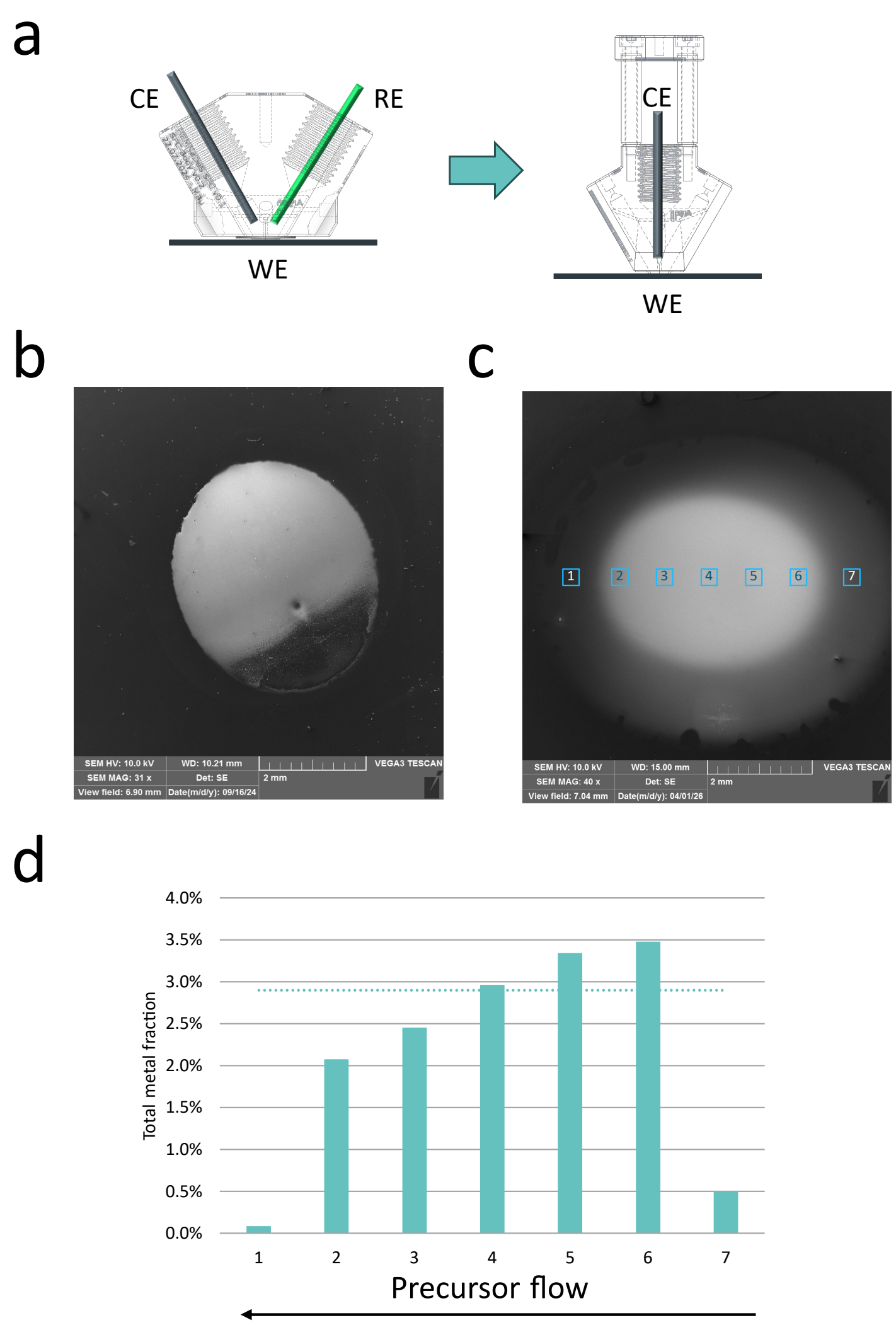

Figure S2: Electrode positioning and homogeneity of spot.
a) Change in SFC design. **b)** A spot synthesized with a cell with oblique CE and RE, showing greater coverage toward the side of CE. **c)** A spot synthesized with a cell with the CE sitting perpendicular above the sample, and the RE in a separate container. **d)** Coverage as determined by EDX, on the spots as numbered in **c**. Only spots 2~6 (in the central bright area in **c)**) lie under the opening and are subject to direct electrical current. Dashed line in **d)** represents the average of these spots.

4. Calculation of reduction potentials

The concentration of Cl^- from the dissociation of 2.5 mM of $[AuCl_4]^-$, $[PdCl_4]^{2-}$ and $[PtCl_6]^{2-}$ is calculated from the cumulative stability constants of those complex ions (**Table S2**). The concentration of Cl^- in mixtures from the three precursors with a total concentration of 2.5 mM should be within the range between 0.00015 and 0.0038 mol L^{-1}. The reduction potentials calculated from the Nernst equation (S2) with T = 25 °C and $[Cl^-]$ = 0.00015 or 0.0038 mol L^{-1} represent their maximal possible range in different precursor mixtures.

$$E = E^\circ - \frac{RT}{nF}\ln Q \tag{S2}$$

Complex	log β_1	log β_2	log β_3	log β_4	log β_5	log β_6	$[Cl^-]$ / mol L^{-1}	Ref.
[a]$[AuCl_4]^-$	8.51	16.57	23.6	29.6	-	-	0.00015	[1]
[b]$[PdCl_4]^{2-}$	4.47	7.74	10.2	11.5	-	-	0.0038	[1]
$[PtCl_6]^{2-}$	4.8	8.6	11.3	13	16.7	19	0.0033	[2]

Table S2: Cumulative stability constants and calculated Cl^- concentrations.

Concentrations in a solution with 2.5 mM of the respective precursors arec calculated. a: Values at 20 °C with an ionic strength of 0 mol L^{-1}; β_1 is for $[M(OH)_3L]/[M(OH)_4][H][L]$, *etc.* b: Values at 25 °C with an ionic strength of 1.0 mol L^{-1}.

5. Nanoparticle morphology

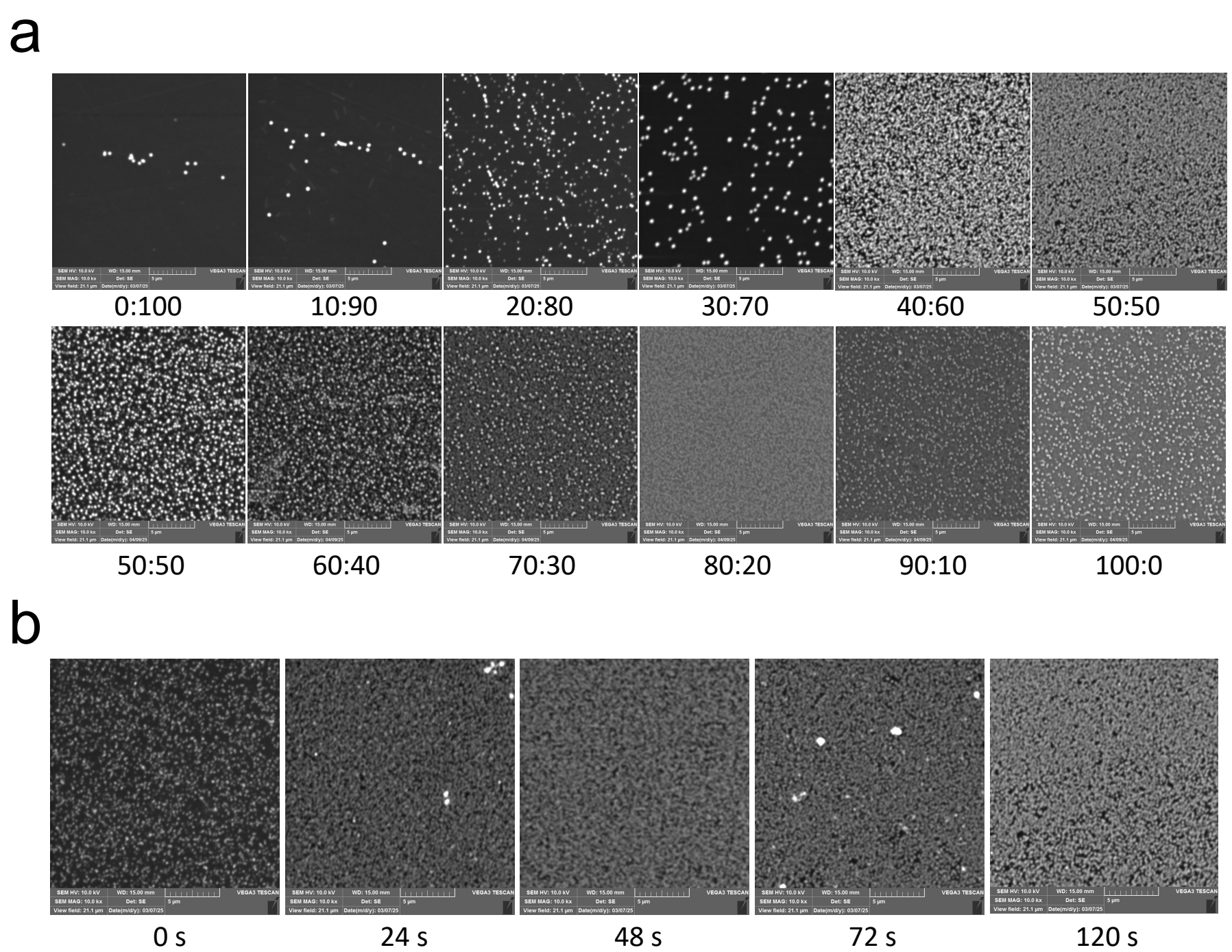

Figure S3: SEM images of nanoparticles from different synthesis conditions.
a) Duty cycle and particle morphology. **b)** Synthesis time and particle morphology. The experiments were conducted with equimolar precursors.

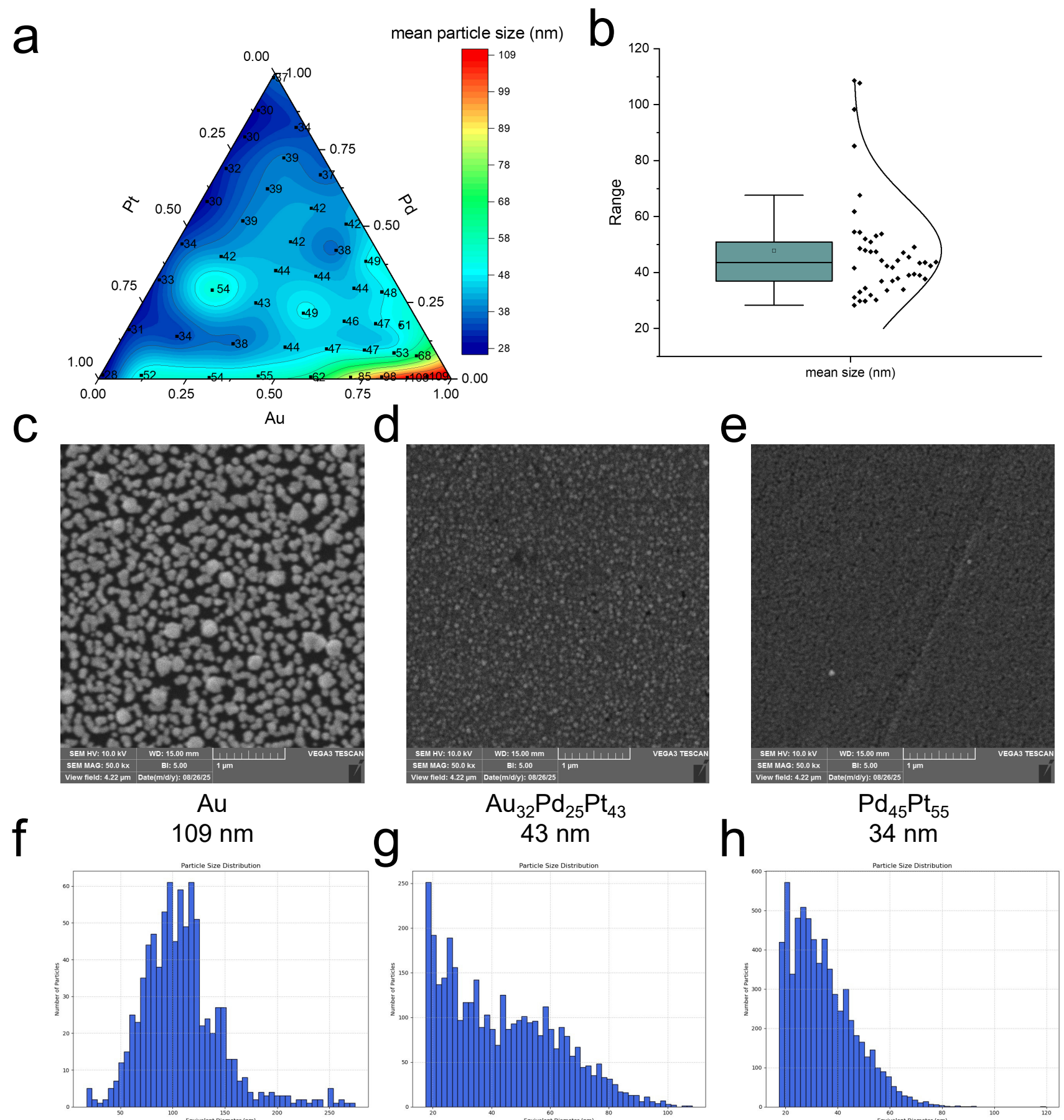

Figure S4: Nanoparticle size from SEM images.
a) Mean particle size of different compositions. **b)** Distribution plot of mean particle size. **c, d, e)** Representative images of three samples with their composition and mean particle size. **f, g, h)** Size distribution of the representative samples.

The particle size was automatically determined by a script modified from Gautam *et al.*[3] (see supplementary data), where the SEM images were thresholded and segmented by a watershed algorithm. The minimum peak distance was set at 20 nm, and the mimimum particle size at 200 nm^2 to filter imaging noise.

6. Transmission electron microscopy

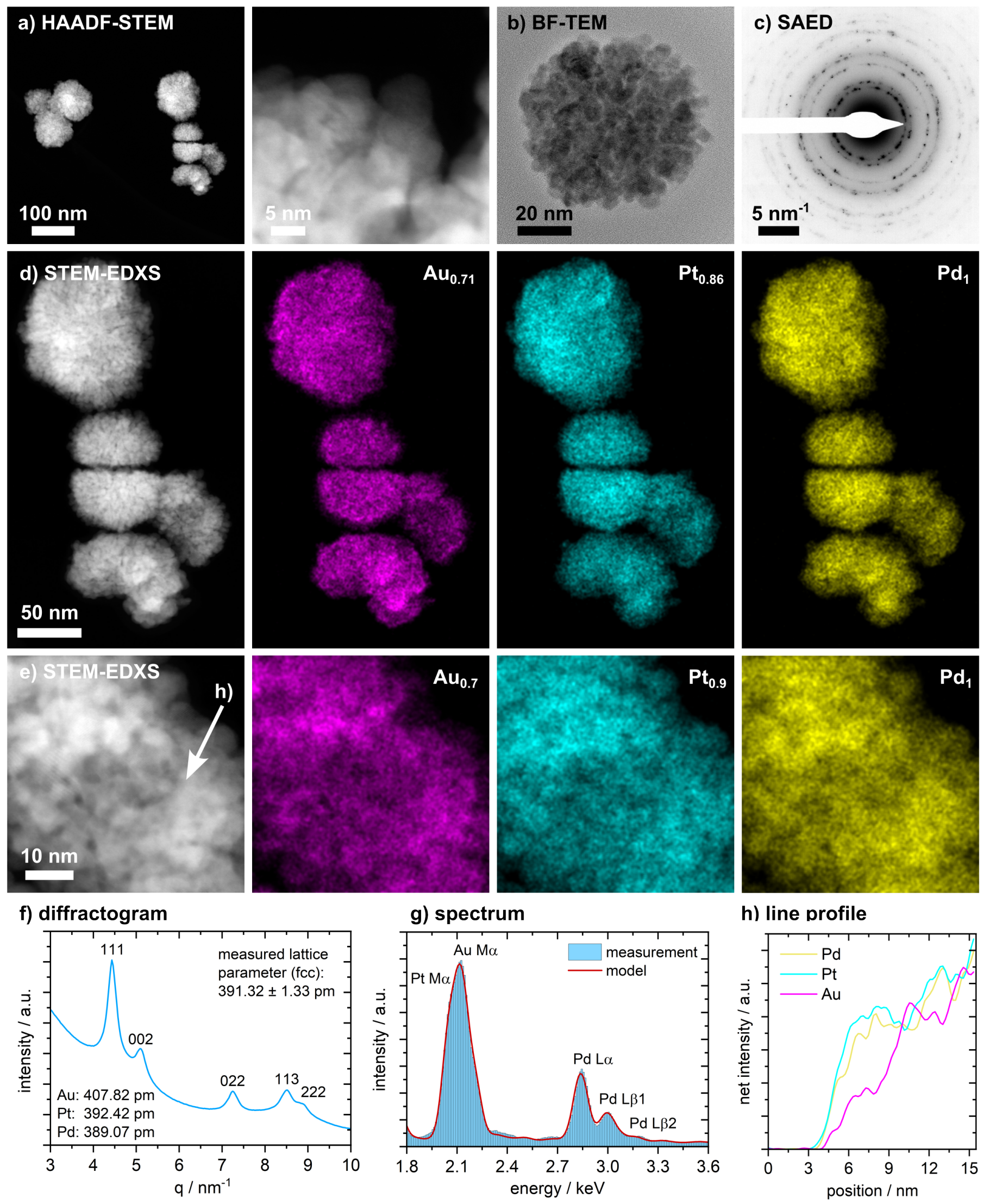

Figure S5: TEM analysis of a three-element sample.
a, b) HAADF-STEM and high-resolution TEM images of the ternary Au-Pd-Pt alloy. **c, f)** SAED pattern and corresponding diffractogram. **d, e, g)** EDX spectrum images and corresponding EDX spectrum showing Pt, Au, and Pd emission lines. **h)** elemental line profile as shown in **e)** revealing a slight depletion of Au at the particle surface

Transmission electron microscopy (TEM) analyses have been performed using a double aberration corrected Titan^3 Themis 60-300 (S)TEM from Thermo Fisher

Scientific which was operated at a primary electron energy of 300 keV and a beam current of roughly 150 pA. The microscope was used for imaging employing image corrected bright-field TEM and probe corrected high-angle annular dark field scanning TEM (HAADF-STEM) as well as for crystal structure analysis using selected area electron diffraction (SAED). STEM energy dispersive X-ray spectrum images (STEM-EDXS) were acquired with a Super-X EDS detector.

Selected area electron diffraction (SAED) of the ternary alloy shows an fcc structure with a lattice parameter of 391.32 $\pm$ 1.33 pm which is very close to that of Pt (392.42 pm) and Pd (389.07 pm). Moreover, scanning transmission electron microscopy energy dispersive X-ray spectroscopy (STEM-EDXS) reveals a homogeneous distribution of Au, Pt, and Pd whereas a slight Pt/Pd enrichment and Au depletion at the surface becomes apparent from an EDX line profile. The elemental composition is on the order of $Au_{0.7}Pd_1Pt_{0.9}$ ($Au_{27}Pd_{38}Pt_{35}$) with an error of approximately 18%, which agrees with the SEM-EDX composition of $Au_{24}Pd_{41}Pt_{35}$. High-angle annular dark field STEM (HAADF-STEM) shows an aggregate particle size of 71.5 $\pm$ 23.0 nm (n = 14) and bright field TEM a crystallite size of 4.8 $\pm$ 1.1 nm (n = 36) which agrees well with 4.1 nm obtained from the peak width of the 111 peak (Scherrer equation).

7. X-ray diffraction

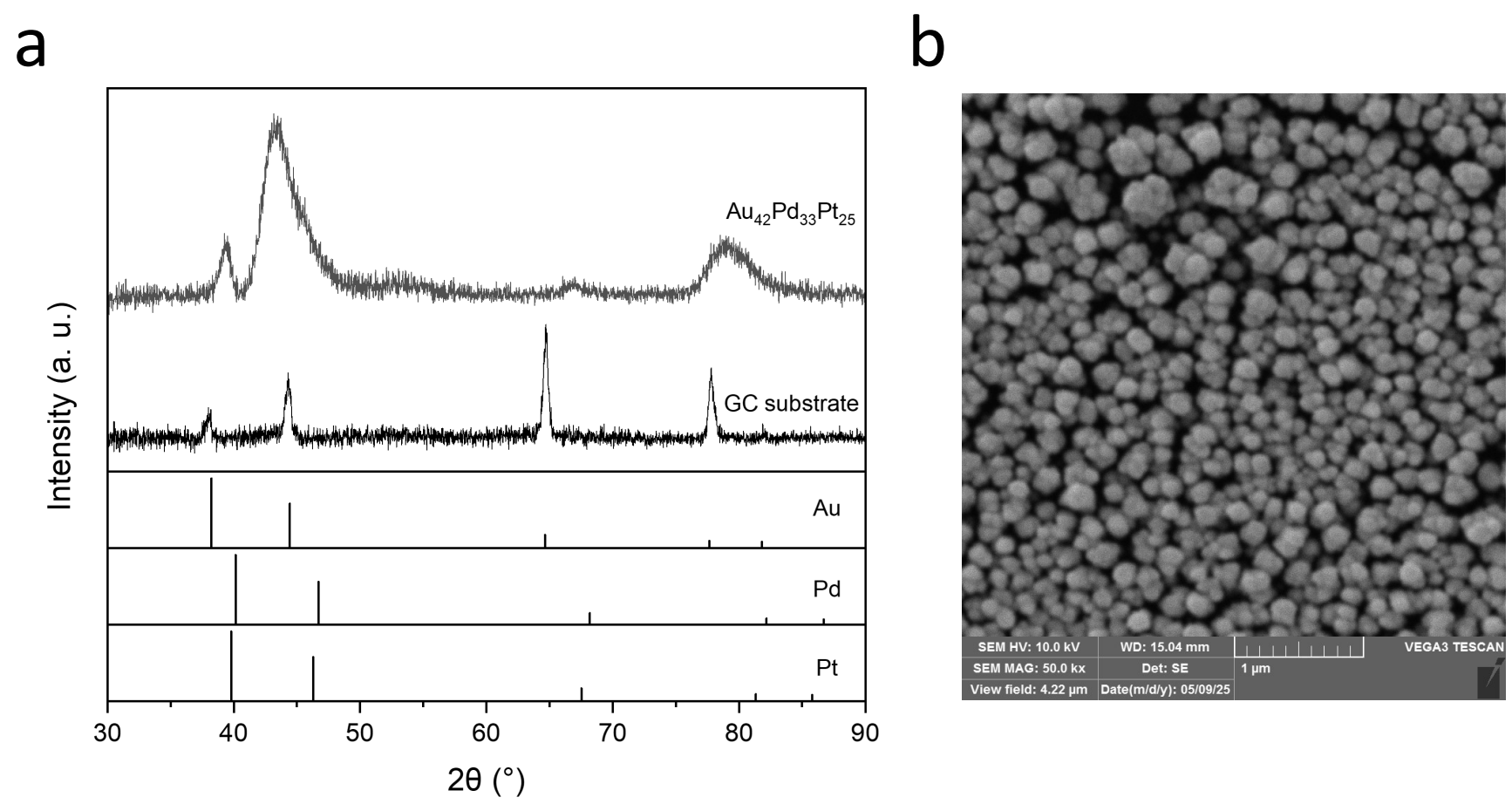

Figure S6: XRD patterns of a three-element sample.
a) XRD patterns with peak positions of the individual elements (ICSD-52249~52251 (ICSD release 2026.2)[4]). **b)** SEM image of the sample.

X-ray diffraction (XRD) was measured on a Bruker D8 Advance with glazing incidence diffraction (Cu anode, 1.5406 Å, step time 6 s, step size 0.02°). The sample was deposited on a 10 mm $\times$ 10 mm glassy carbon substrate, from an equimolar mixture of the Au, Pd and Pt precursors, under the usual synthesis conditions but for 20 min (10 times), in order to get enough loading for the XRD analysis. The composition measured by SEM-EDX was $Au_{42}Pd_{33}Pt_{25}$. The diffractogram shows an fcc structure with lattice parameter of 402 pm, determined from the {311} peak position of $2\theta = 79.0°$.

8. Composition confidence

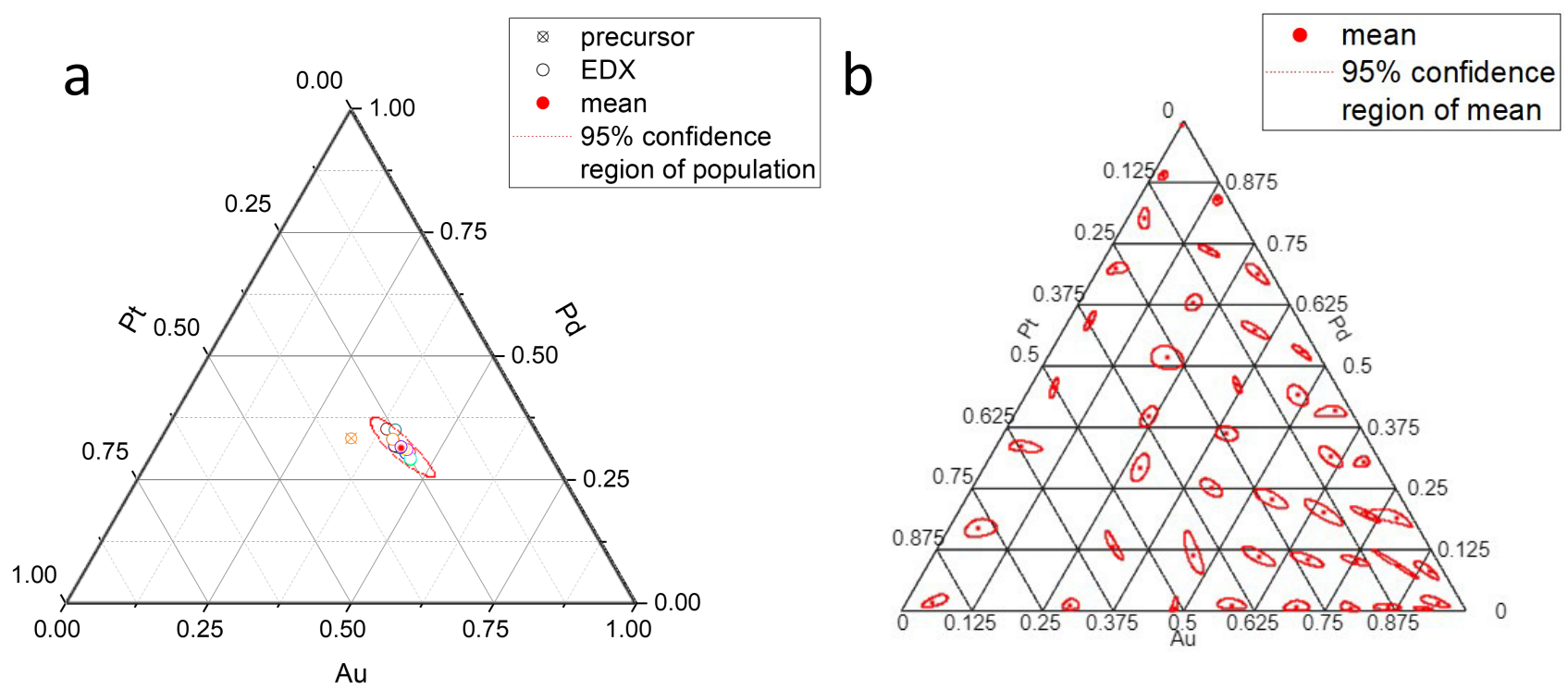

Figure S7: Confidence regions of EDX composition
a) EDX-composition of 15 repeated synthesis from an equimolar precursor, with the 95%-confidence region of population. **b)**95%-Confidence regions of the mean of the 45 samples in the activity dataset, formed by 6 repeated EDX measurements on each sample.

The uncertainty of composition measurement can be represented as confidence regions. A set of measured compositions $\{x_{Au}, x_{Pd}, x_{Pt}\}$ are transformed by additive log-ratio transformation into a two-dimensional space with the basis:[5]

$$\{y_1, y_2\} = \{log(x_{Au}/x_{Pt}), log(x_{Pd}/x_{Pt})\}$$

The arithmetic mean of the vectors in the log-ratio space gives the mean composition, which is equivalent to the geometric mean in the ternary simplex. A confidence ellipse of population representing the 95%-probable range of the next measurement, or a confidence ellipse of mean representing the 95%-probable range of the mean, is determined by Hotelling's t-squared statistic. The confidence ellipses are transformed back into the ternary simplex and plotted in **Figure S7**. The MATLAB script for the analysis above is included in the supplementary data.

9. Composition cross-validation

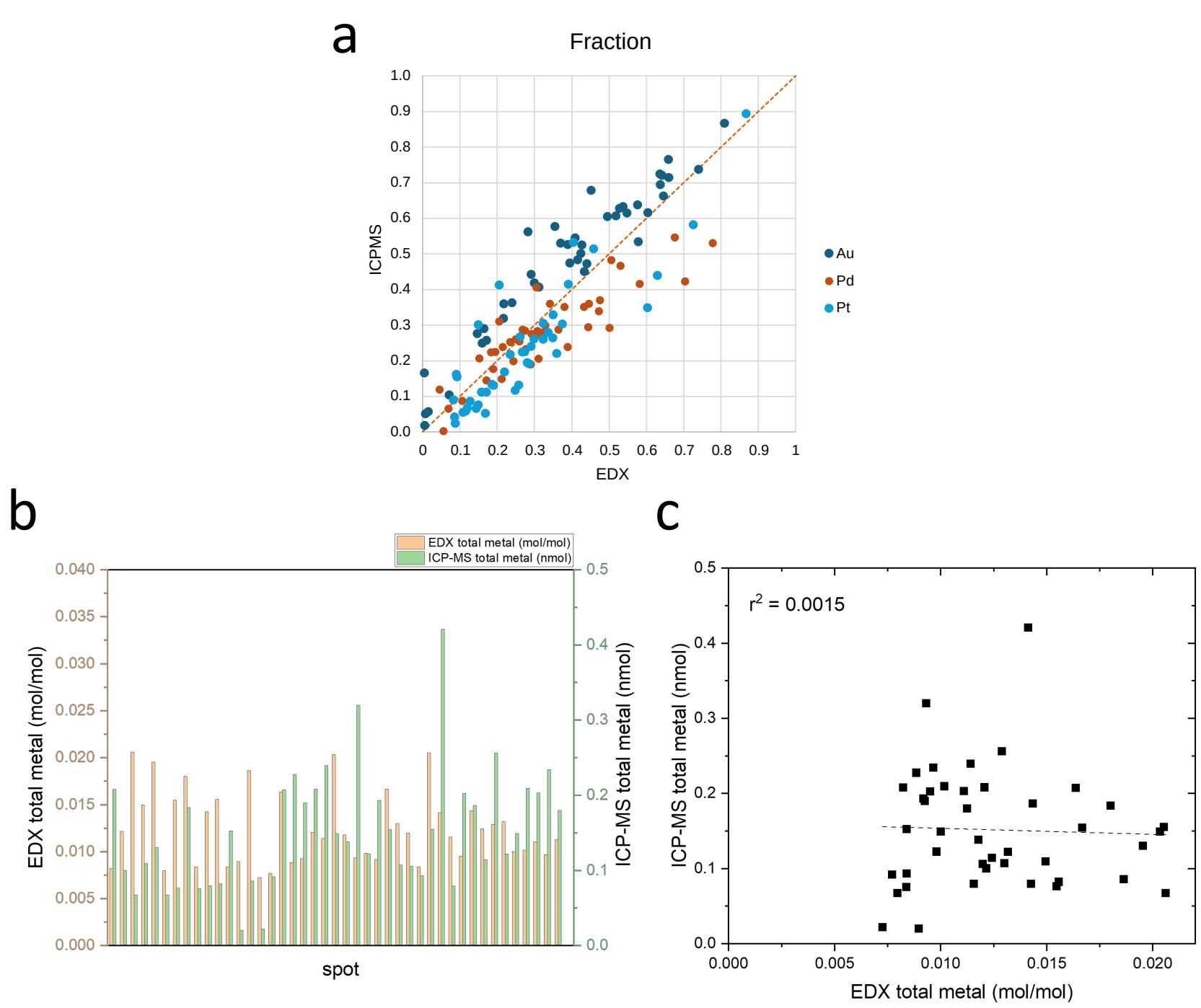

Figure S8: Composition determination by ICP-MS and SEM-EDX
a) ICP-MS *vs* SEM-EDX compositions of 43 samples. **b)** Side-by-side comparison of mass loading determined by EDX (as the total mole fraction of the metals), and by ICP-MS (as the sum of the amounts of substance). **c)** Correlation between ICP-MS and EDX mass loadings.

	β	R^2
Au	1.16	0.98
Pd	0.86	0.92
Pt	0.81	0.95

Table S3: Linear regression between ICP-MS and EDX compositions.
β: Regression coefficient. R^2: Coefficient of determination. The model function is $y = \beta x$, where y represents ICP-MS fractions and x represents EDX fractions of an element.

Procedure of ICP-MS elemental analysis: scrub the sample off the substrate with a cotton swab and immerse in 1 ml of aqua regia, until the metal particles completely dissolve. Take 0.1 ml of the solution and dilute to 10 ml with deionized water. Analyze the solution with ICP-MS.

10. Electrochemical characterization

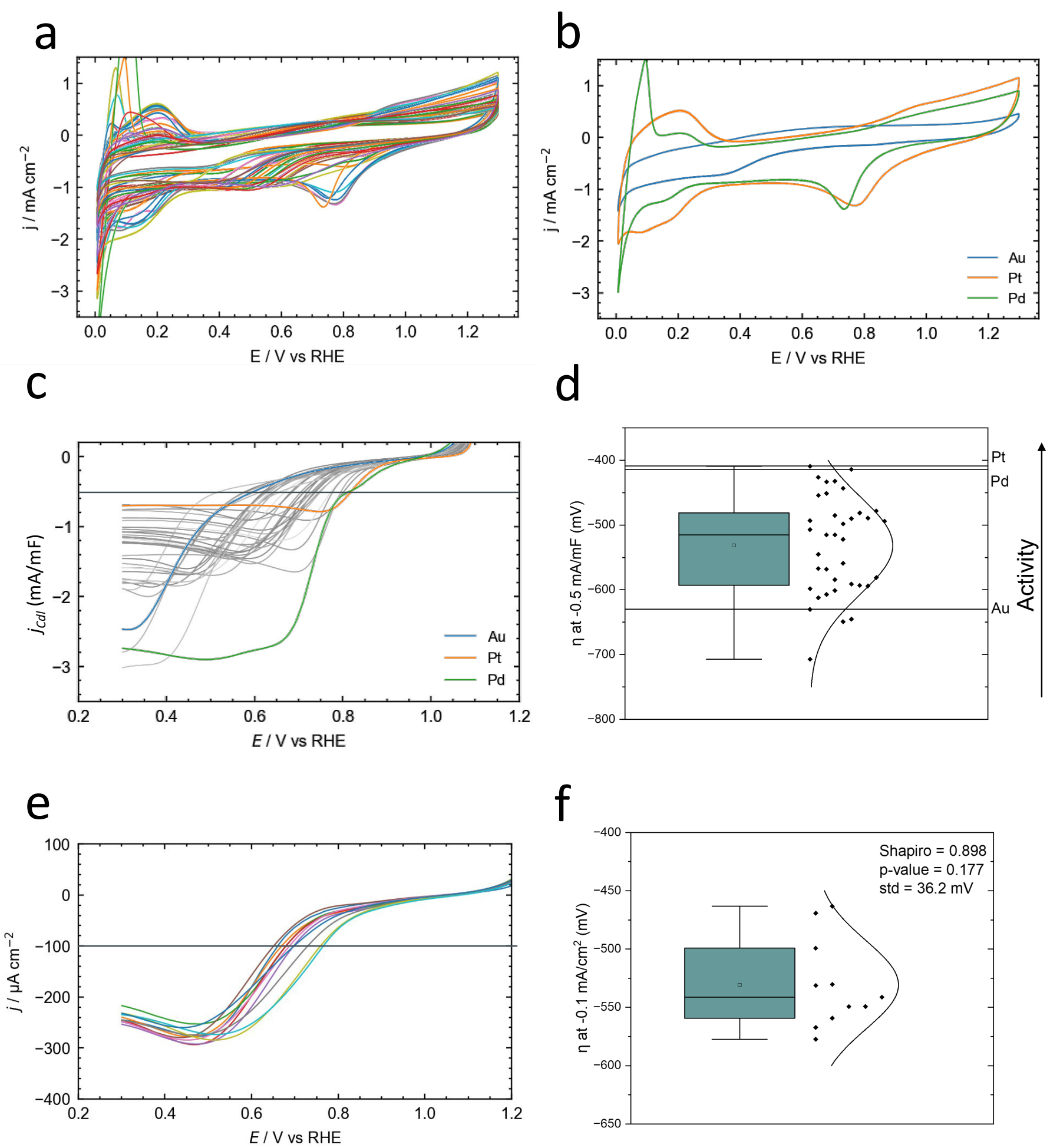

Figure S9: Electrochemical characterization during activity measurement.
a) CV scans during conditioning of all samples, scan rate: 100 mV/s. **b)** CV of the single-element samples. **c)** LSV scans of all samples, with ECSA-normalization. The single-element samples are highlighted. **d)** Box and distribution plot of ECSA-normalized activity. Activities of the single-element samples are additionally shown as horizontal lines. **e, f)** Repeated synthesis and test of the equimolar composition by 11 times. **e)** LSV curves. **f)** Distribution of the overpotential at -0.1 mA/cm^2.

In the cyclic voltammograms of Pt and Pd, the under-potential deposition of hydrogen is visible under 0.3 V. A feature corresponding to the reduction of Pt/Pd-

oxide is visible at 0.7~0.8 V during negative scan, which also occurs in the LSV curve of some compositions.[6, 7]

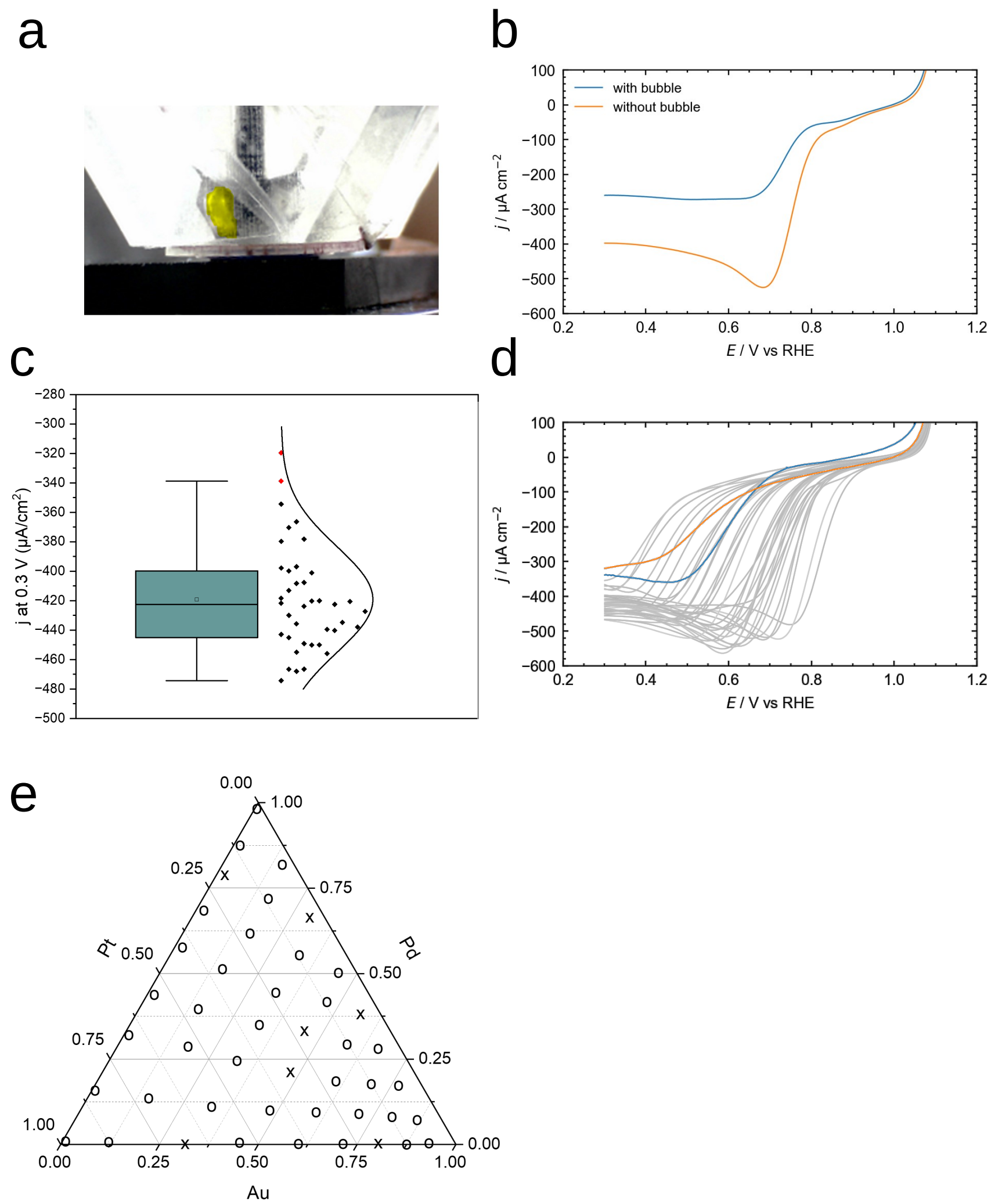

Figure S10: Effect of bubble on LSV measurement.
a) SFC with a highlighted bubble partially blocking the pathway between the WE and the RE. **b)** Effect of a bubble on the LSV of the same sample, causing the limiting current density to shift upwards. In case of complete obstruction, the sample is destroyed by potential overload. **c)** Box plot of the distribution of the limiting current density at 0.3 V, the colored points being outliers. **d)** LSV curves with the outliers affected by a bubble marked by colors. **e)** Excluded samples because of partial or total blockage by a bubble are marked with an x.

11. Double layer capacitance measurement

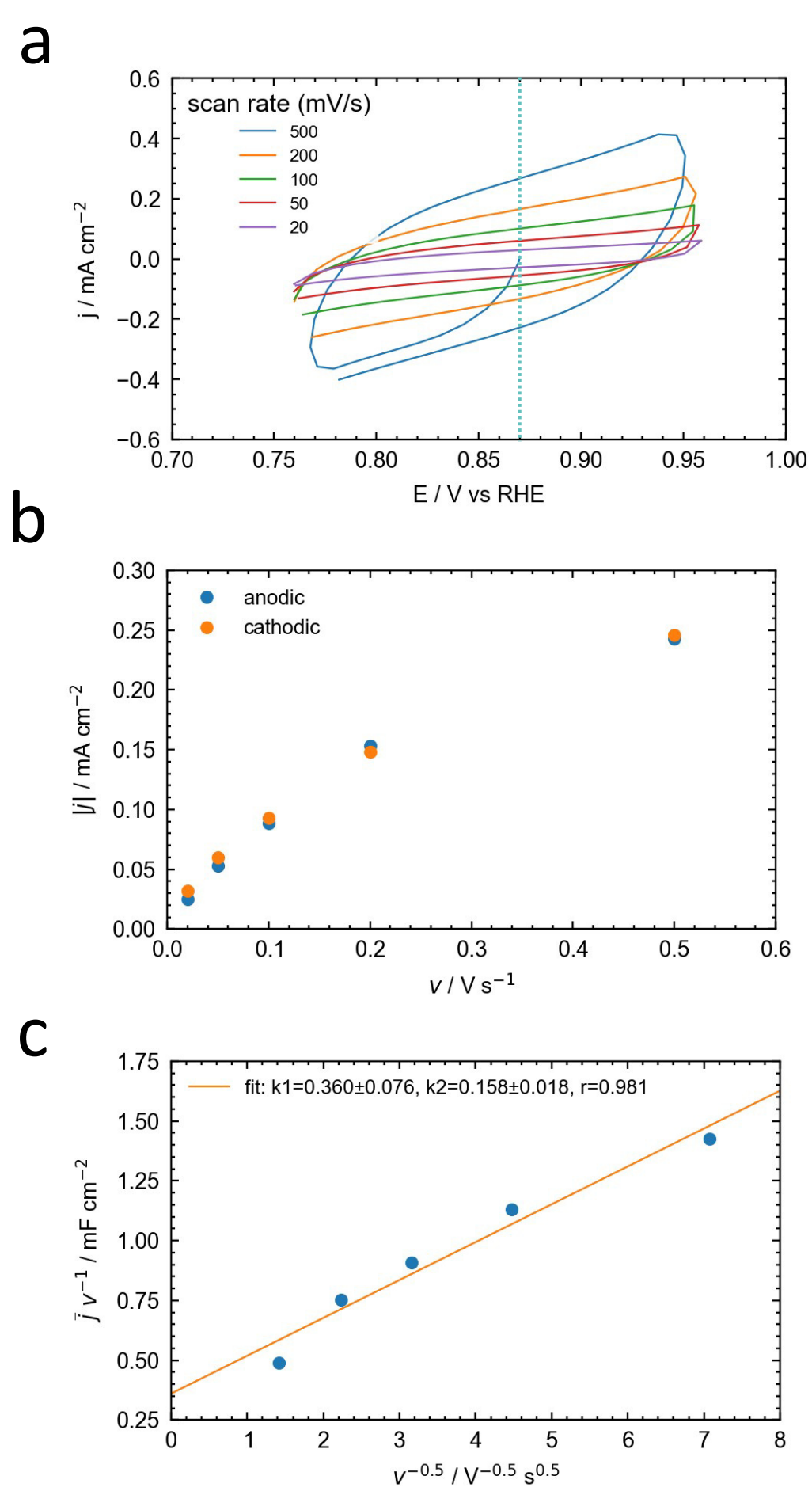

Figure S11: Example of double layer capacitance measurement.
a) CV scans of ±0.1 V around OCP (indicated by dashed line) on $Au_{32}Pd_{45}Pt_{23}$. **b)** Absolute values of current density at OCP during anodic and cathodic scans, plotted against scan rate ν. **c)** Average current density $\bar{j} = (|j_a| + |j_c|)/2$ divided by ν, plotted against $\nu^{-0.5}$. The linear regression intercept $k1$ is taken as the C_{dl}. The Python script is available in the supplementary data.

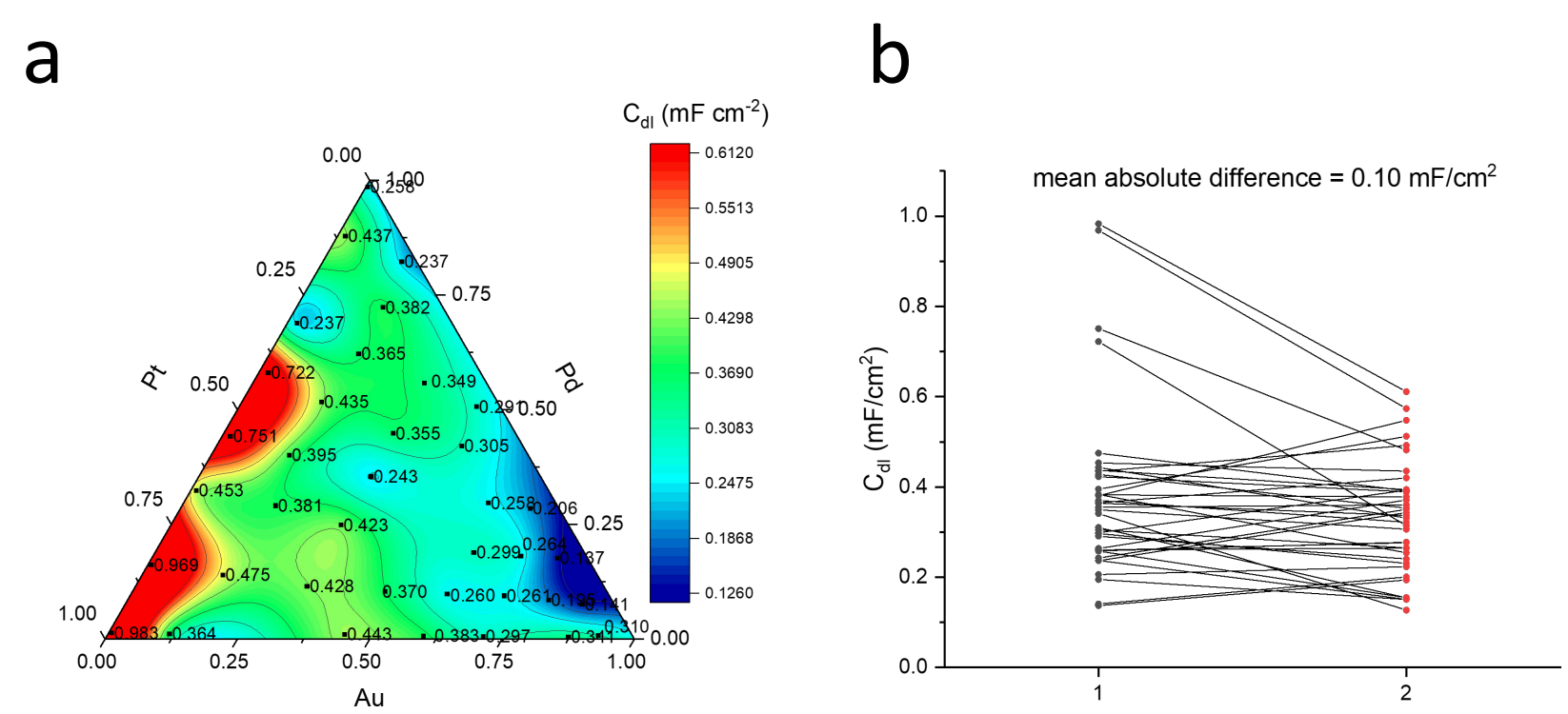

Figure S12: Measurement uncertainty of C_{dl}

a) Results of repeated measurement of C_{dl} on 38 samples. **b)** Change between two measurements. The results was extracted from two suits of C_{dl} - activity measurements with an LSV and a conditioning-CV between them, therefore the uncertainty in C_{dl} might be exaggerated.

12. Stability measurement

Technique	Potential / V_{RHE}	Scan rate / mV s^{-1}	Cycles	Duration / s
OCP	-	-	-	300
CV	0.05~1.3	100	4	110
OCP	-	-	-	30
CV*	OCP±0.1	500~20	1 each	40
LSV	1.1~0.3	10	1	80
OCP	-	-	-	120
Pulse	0.6, 1.4	3 s each step	200	1200
OCP	-	-	-	120
CV	0.05~1.3	100	4	110
OCP	-	-	-	30
CV*	OCP±0.1	500~20	1 each	40
LSV	1.1~0.3	10	1	80
CV	0.3~1.4	10	1	220
OCP	-	-	-	120

Table S4: Electrochemical protocol for stability study.
OCP: open circuit potential, CV: cyclovoltammetry, LSV: linear sweep voltammetry. *Five cycles of CV for measuring the double-layer capacitance, as described in the main text, section 2.1.

Sample	Precursor composition			EDX composition			Dissolution (nmol)				Dissolution potential		
	Au	Pd	Pt	Au	Pd	Pt	Au	Pd	Pt	Total	Au	Pd	Pt
1	1.000	0.000	0.000	0.910	0.011	0.075	0.006	0.001	0.001	0.007	an	-	-
2	0.000	0.125	0.875	0.017	0.155	0.818	0.005	0.111	0.442	0.558	-	an	an+cat
3	0.000	0.625	0.375	0.012	0.684	0.301	0.000	0.536	0.229	0.765	-	cat	cat
4	0.000	1.000	0.000	0.003	0.989	0.007	0.000	3.458	0.016	3.475	-	an	-
5	0.250	0.750	0.000	0.286	0.683	0.029	0.002	0.258	0.010	0.270	-	an	-
6	0.375	0.625	0.000	0.409	0.554	0.036	0.002	0.193	0.005	0.199	an	an	-
7	0.750	0.250	0.000	0.739	0.193	0.067	0.019	0.120	0.018	0.157	an	an	-
8	0.125	0.250	0.625	0.132	0.280	0.587	0.007	0.209	0.421	0.637	an	an	an
9	0.125	0.500	0.375	0.177	0.500	0.321	0.005	0.237	0.231	0.473	an	an	an
10	0.375	0.250	0.375	0.444	0.219	0.334	0.008	0.092	0.140	0.240	an	an	an+cat
11	0.000	0.375	0.625	0.011	0.429	0.557	0.001	0.901	0.332	1.234	-	an	cat
12	0.625	0.000	0.375	0.687	0.006	0.305	0.001	0.005	0.145	0.151	-	-	cat
13	0.250	0.000	0.750	0.324	0.012	0.663	0.002	0.007	0.292	0.301	-	-	cat
14	0.125	0.000	0.875	0.125	0.016	0.855	0.005	0.003	0.418	0.427	an	-	an+cat
15	0.000	0.000	1.000	0.017	0.019	0.961	0.002	0.006	0.290	0.299	-	-	cat

Table S5: Composition of the samples tested for stability and their dissolution.
Dissolution potential lists the main occurence of dissolution signals during the last CV of the stability protocol. *an(odic)* indicates dissolution around the highest potential of 1.4 V, *cat(hodic)* indicates dissolution that peaks later, during the descending of potential.

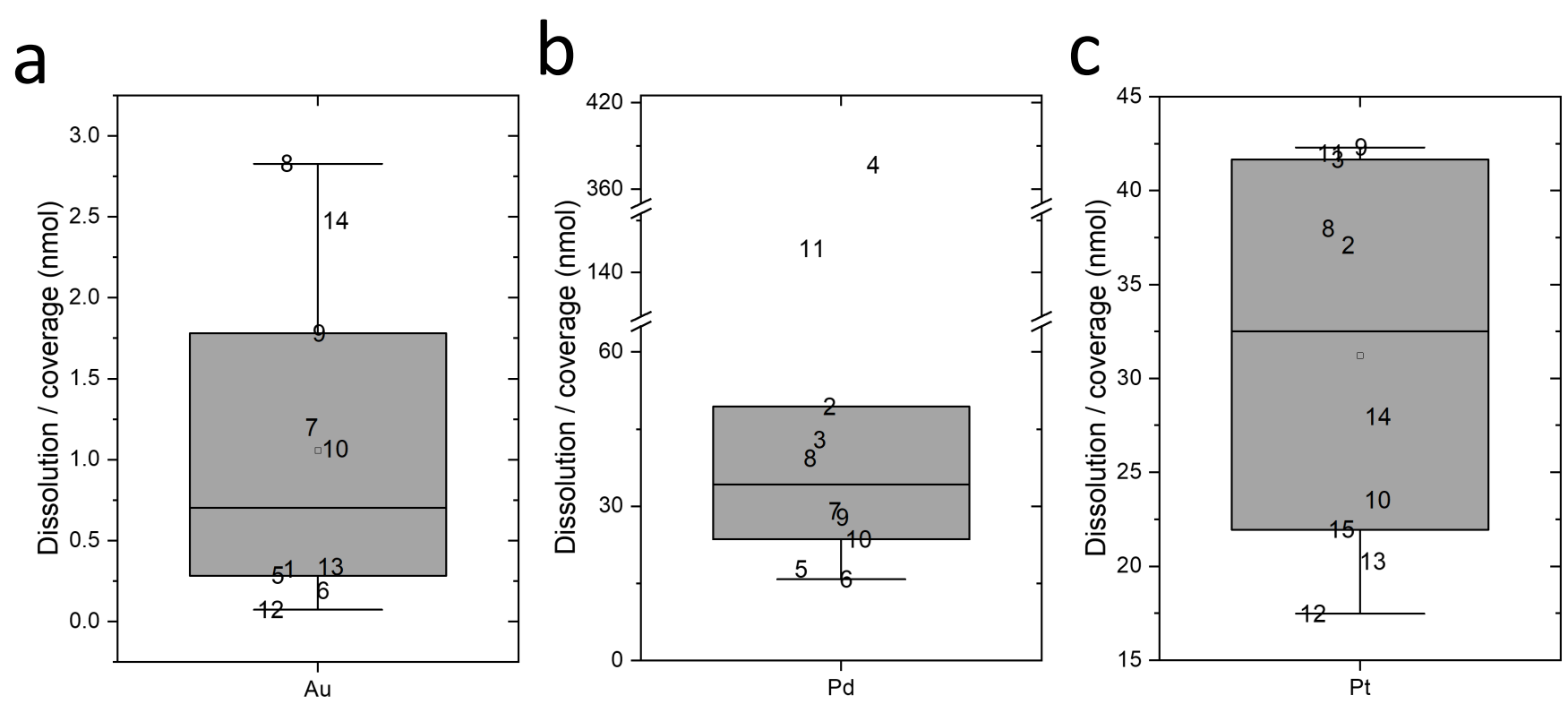

Figure S13: Distribution of relative dissolution for each element.
The y-axis is the dissolution of each element divided by the EDX-coverage of that element. The grey box contains the 25% to 75% percentile of the data. The horizontal line inside the box is the median, and the hollow square is the average. Numbering of samples corresponds to **Table S5**. Samples without certain element in the precursor are omitted in the graph of that element, to avoid division by a small number. In **b)**, out of 6 samples containing Au, 5 have lower dissolution than the median. In **c)**, out of 6 samples containing Au, 4 have lower dissolution than the median.

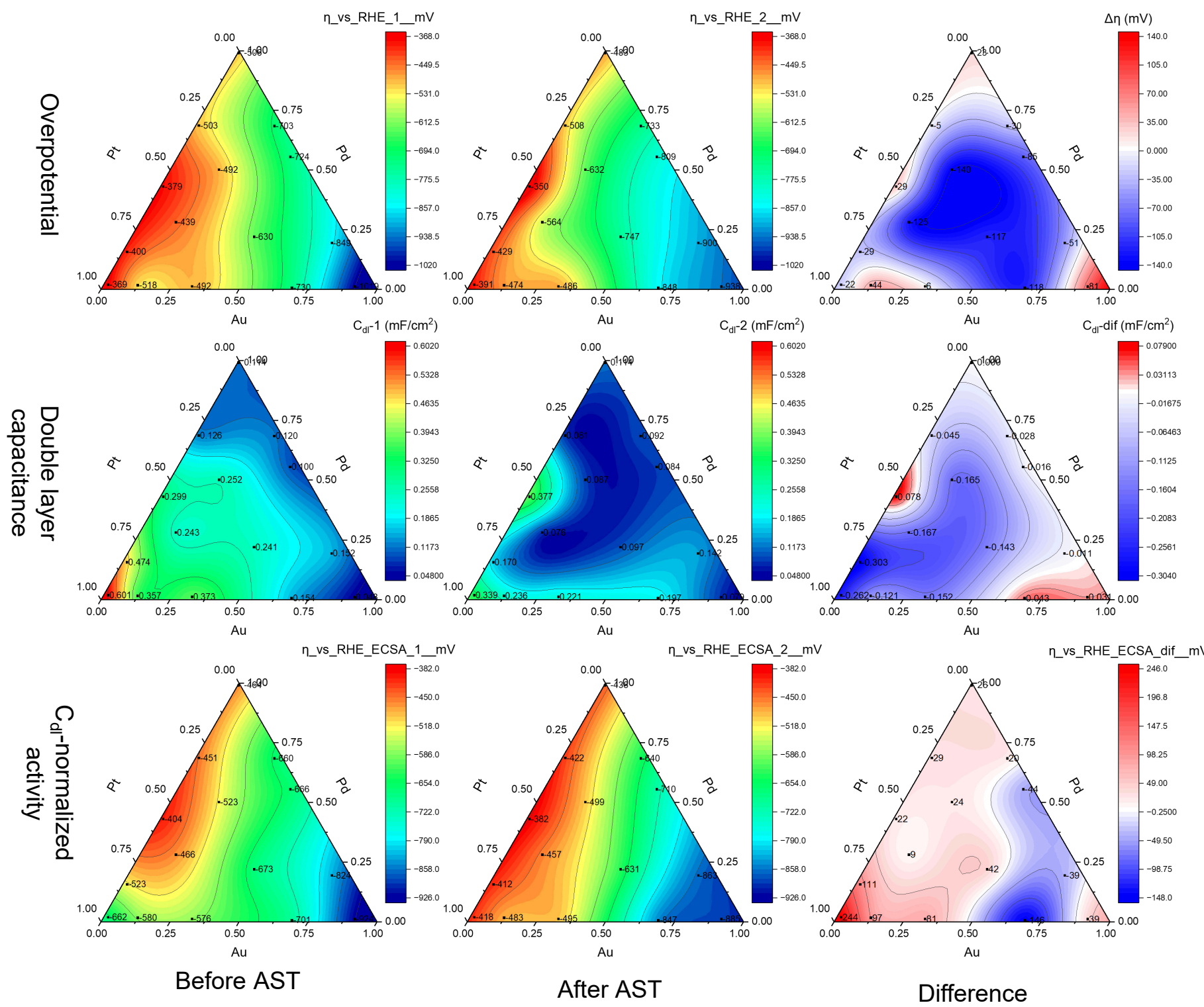

Figure S14: Before-and-afters of the ORR overpotential, ECSA and ECSA-normalized activity.

13. Multiobjective optimization

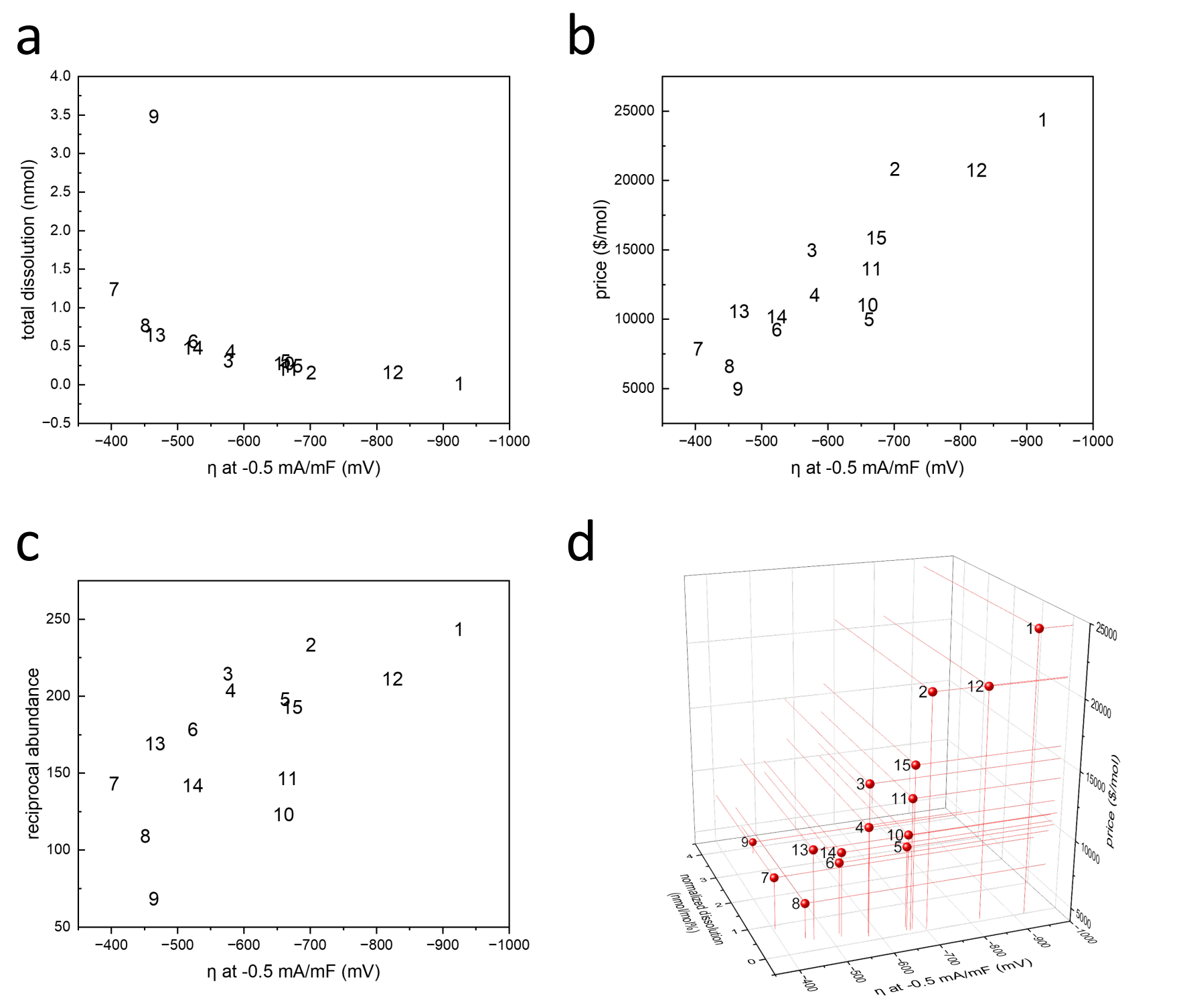

Figure S15: Pareto fronts of the compositions.
a) Total dissolution amount and activity. **b)** Cost of the material[8] and activity. **c)** Metric based on the reciprocal of the abundances of the elements in Earth's crust[9], averaged by alloy composition. **a), b), c)**: Lower left is better. **d)** 3-dimensional representation between price, activity and stability. The compositions of the numbered spots are represented in **Table S5**.

14. Gaussian process models

n	without white kernel			with white kernel			
	MAE (mV)	constant	length scale	MAE (mV)	constant	length scale	noise variance
**3**	99.146	316	2.11	75.702	316	1.00E+05	3.99E+04
**4**	58.906	316	1.88	54.28	316	4.13	1.69E+04
**5**	47.472	316	1.77	45.017	316	3.36	8.94E+03
**6**	48.922	316	1.94	42.69	316	2.91	4.13E+03
**7**	48.936	316	2.16	48.936	316	2.16	0.000129
**8**	48.754	316	2.34	48.754	316	2.34	6.81E-05
**9**	47.416	316	2.35	47.416	316	2.35	4.74E-05
**10**	47.11	316	2.47	47.11	316	2.47	2.37E-05
**11**	44.281	316	2.08	44.281	316	2.08	0.000197
**12**	43.338	316	1.74	43.295	316	1.74	9.41
**13**	43.204	316	1.82	43.204	316	1.82	0.000201
**14**	43.305	316	1.88	43.305	316	1.88	4.03E-05
**15**	44.652	316	1.6	42.836	316	1.6	272

Table S6: Fitted hyperparameters of GP with and without white kernel to represent noise. MAE is measured against a 37-sample ground truth.

The noise in the data can be estimated by adding a noise term $\sigma_n^2 \delta_{pq}$, with the noise variance σ_n^2 as hyperparameter.[10] However, this additional hyperparameter was difficult to estimate from our dataset. The noise variance turned out to be overly large for small sample sizes that even led to the fit not converging (n=3~6), or very close to zero so that it had no effect on the model for intermediate sample sizes (n=7~14). For the final sample set (n=15), the prediction of the activity model was similar with or without the noise term (Fig. S16f). Therefore, no noise term was included in the GP model.

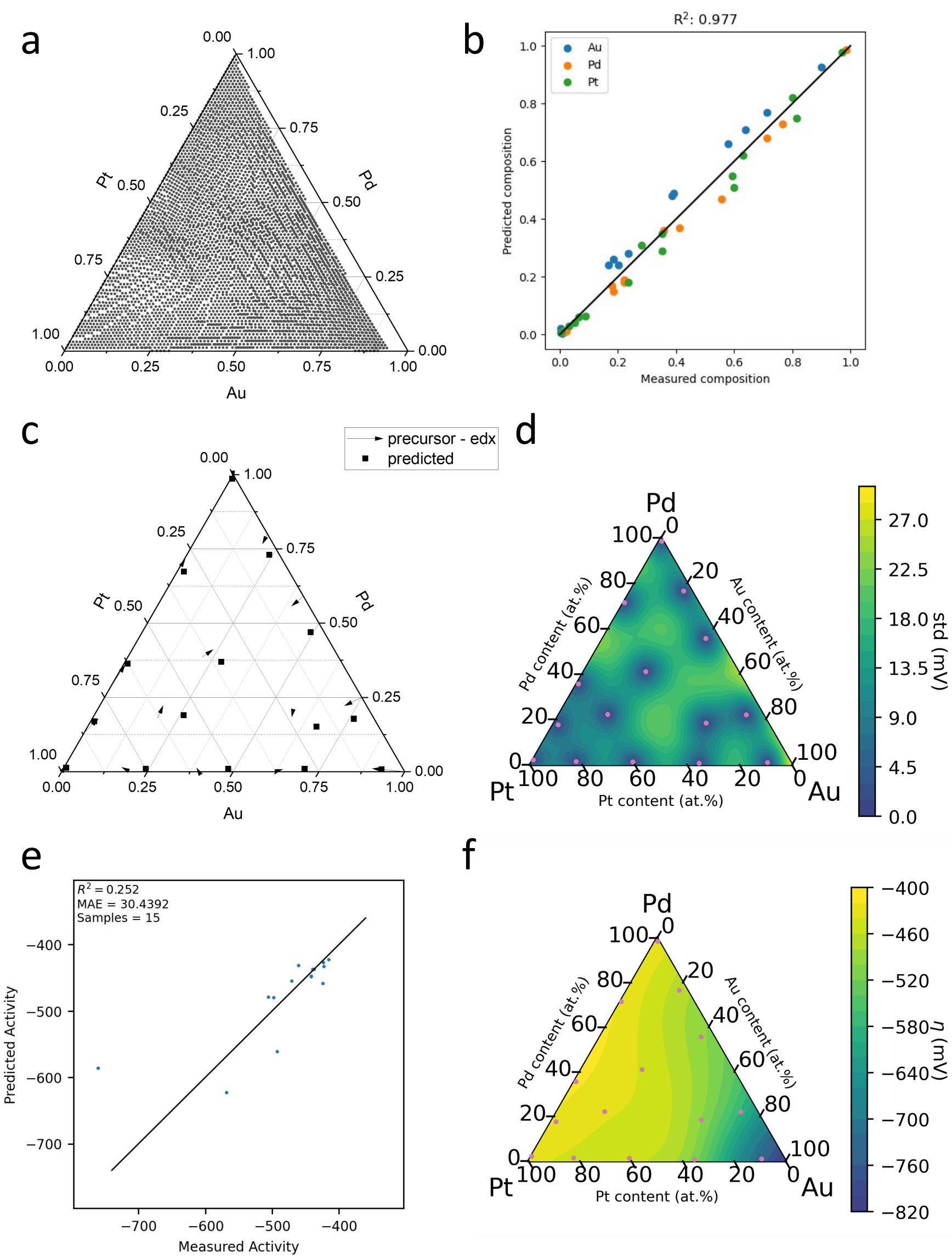

Figure S16: Outputs of Gaussian Process models.
a) Prediction of all possible EDX-compositions from precursor-compositions, by the composition model. **b)** Performance of the composition model on 15 samples. **c)** Predicted and measured EDX-compositions of 15 samples. **d)** Uncertainty of prediction of the activity model with 15 samples. **e)** Validation plot of the activity model using the leave-one-out method. **f)** Prediction of the GP model that includes a noise term for the activity.

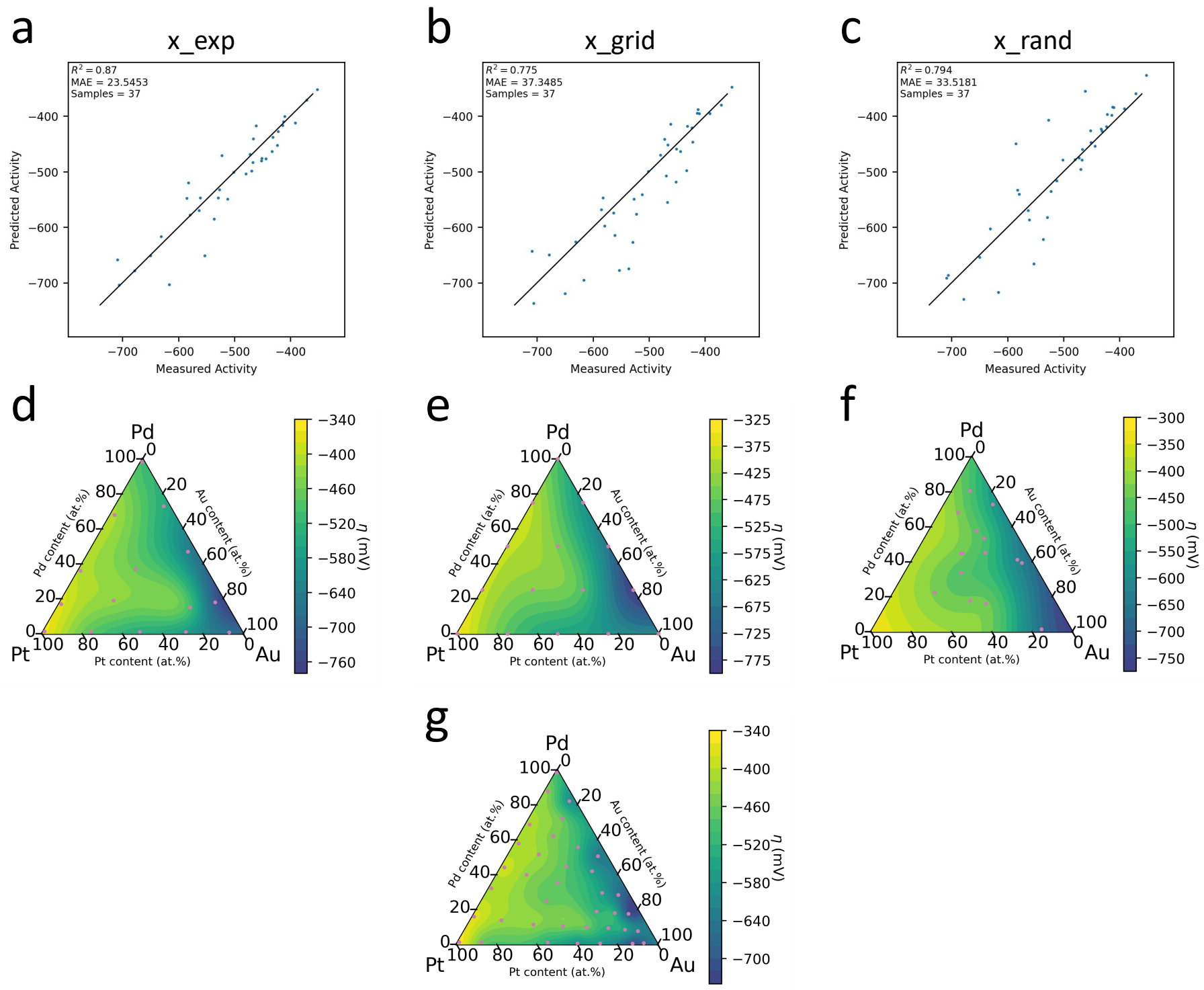

Figure S17: Comparison of sampling methods for GP.
a, b, c) Prediction errors of the GP models on the activites of 37 experimental samples. **d, e, f)** GP models trained with 15 samples drawn from different sampling methods (shown as points). The activities of the samples are provided by a GP model **g)** trained on the experimental data as the ground truth.

x_exp: the samples suggested by Bayesian active learning, as in main text, section 3.3. x_grid: a uniform grid spaced by 25%. x_rand: random points drawn by the uniform order statistics method.

References

[1] D. R. Burgess, *NIST SRD 46. Critically Selected Stability Constants of Metal Complexes: Version 8.0 for Windows*, 2020, `https://data.nist.gov/od/id/mds2-2154`, DOI: 10.18434/M32154.

[2] Y. Baba, A. Arima, S. Kanemaru, M. Iwakuma and T. Oshima, *JOURNAL OF CHEMICAL ENGINEERING OF JAPAN*, 2011, **44**, 686–691.

[3] D. Gautam, G. K. H. Wiberg, J. Quinson, D. Wang, C. M. Clausen, R. Rohde, R. Klemmt, J. Rossmeisl, E. D. Bøjesen and M. Arenz, *Small Structures*, 2026, **7**, e202500666.

[4] E. Owen and E. Yates, *The London, Edinburgh, and Dublin Philosophical Magazine and Journal of Science*, **15**, 472–488.

[5] J. Aitchison, **44**, 139–160.

[6] R. Sharma, S. Gyergyek and S. M. Andersen, *Applied Catalysis B: Environmental*, 2022, **311**, 121351.
[7] L.-l. Fang, Q. Tao, M.-f. Li, L.-w. Liao, D. Chen and Y.-x. Chen, *Chinese Journal of Chemical Physics*, 2010, **23**, 543–548.
[8] Deutsche Rohstoffagentur, *Preismonitor November 2025*, https://www.deutsche-rohstoffagentur.de/DERA/SharedDocs/Downloads/Preise/Preismonitor/pm-2025-11.pdf, (accessed January 2026).
[9] *CRC Handbook of Chemistry and Physics*, CRC Press, Boca Raton, FL, 2005, pp. 14–14.
[10] Carl Edward Rasmussen and Christopher K. I. Williams, *Gaussian Processes for Machine Learning*, MIT Press, 2006.
